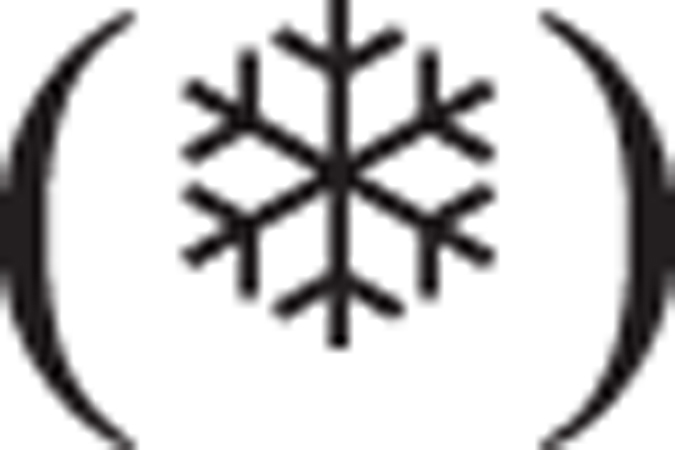# Climate induces seasonality in pneumococcal transmission

**DOI:** 10.1038/srep11344

**Published:** 2015-06-12

**Authors:** Elina Numminen, Claire Chewapreecha, Claudia Turner, David Goldblatt, Francois Nosten, Stephen D. Bentley, Paul Turner, Jukka Corander

**Affiliations:** 1 Department of Mathematics and Statistics, University of Helsinki, Helsinki, Finland; 2 Pathogen Genomics Group, Wellcome Trust Sanger Institute, Hinxton, CB10 1SA, UK; 3 Shoklo Malaria Research Unit, Mahidol-Oxford Tropical Medicine Research Unit, Faculty of Tropical Medicine, Mahidol University, Mae Sot, Thailand; 4 Centre for Tropical Medicine, Nuffield Department of Medicine, University of Oxford, Oxford, UK; 5 Immunobiology Unit, Institute of Child Health, University College London, UK

## Abstract

*Streptococcus pneumoniae* is a significant human pathogen and a leading cause of infant mortality in developing countries. Considerable global variation in the pneumococcal carriage prevalence has been observed and the ecological factors contributing to it are not yet fully understood. We use data from a cohort of infants in Asia to study the effects of climatic conditions on both acquisition and clearance rates of the bacterium, finding significantly higher transmissibility during the cooler and drier months. Conversely, the length of a colonization period is unaffected by the season. Independent carriage data from studies conducted on the African and North American continents suggest similar effects of the climate on the prevalence of this bacterium, which further validates the obtained results. Further studies could be important to replicate the findings and explain the mechanistic role of cooler and dry air in the physiological response to nasopharyngeal acquisition of the pneumococcus.

Young infants are often colonized by pneumococci. While these bacteria typically reside asymptomatically in the nasopharynx, they can invade lungs, brain or blood and cause severe disease, such as pneumonia, meningitis or sepsis. Prevalence of asymptomatic colonization in children varies considerably between countries, while within a country the prevalence appears to persist at approximately a constant level. This becomes especially apparent when looking at the effects of recent vaccination programs. Conjugate vaccines, which protect against the colonization of a subset of pneumococcal strains, are successful in eradicating the targeted strains but not in reducing the overall prevalence of carriage^1^,^2^.

Some colonization studies have indicated that the prevalence within the study population remains stable throughout the year^3^,^4^. However, recent studies conducted in different countries^5^,^6^ suggest that the prevalence of colonization within a population of hosts can display fluctuation over time. Such fluctuations could be purely random, but also due to a number factors such as climate, epidemics of other pathogens, or seasonal behavior of the host population. The same factors could also possibly explain why the prevalence varies significantly between the countries, but the quantitative roles of different contributing factors to variation in transmission and colonization, let alone the seasonality in the incidence of pneumococcal diseases, remain poorly understood^7^.

In this study, we assess the evidence and the possible mechanisms for seasonal differences in the colonization dynamics of the pneumococcus. We analyze longitudinal carriage data using appropriate statistical inference and mathematical modelling tools, showing that transmissibility exhibits significant seasonality; the cool and dry months representing the optimal climate for transmission. Conversely, the average length of a colonization period appears to be constant and independent of the season. Our results may be important for justifying detailed studies of the mechanistic role of cooler and dry climate in the physiological response to nasopharyngeal acquisition of the pneumococcus.

## Results

### Prevalence of colonization in different seasons

**Figure 1:**
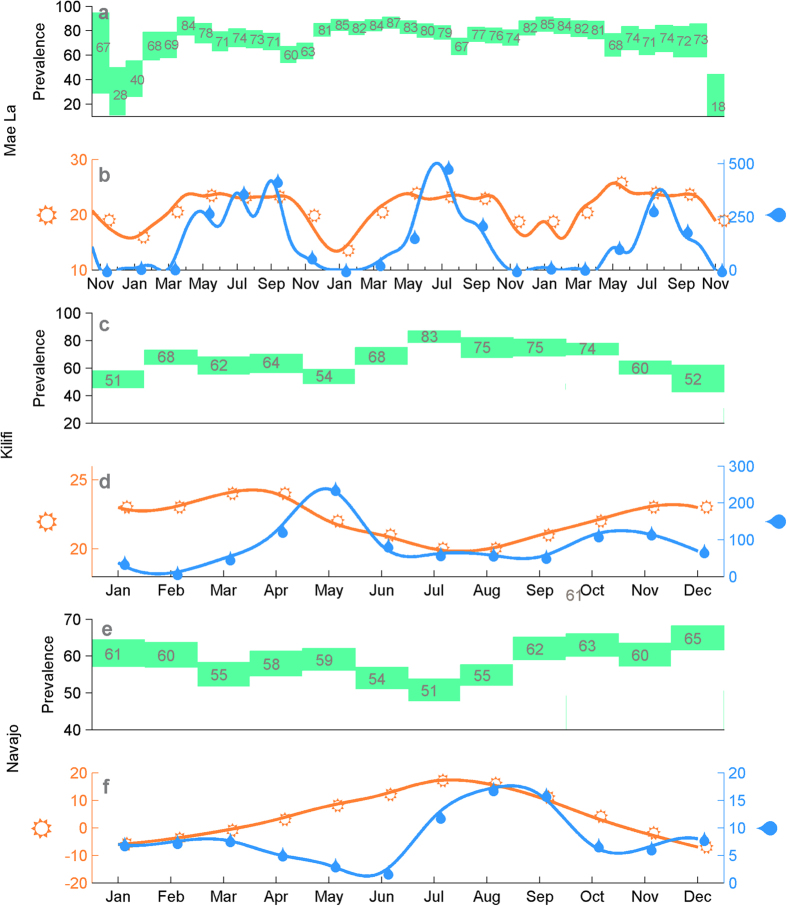
The prevalence of colonization and climate characteristics in three locations: Mae La (Asia), Kilifi (Africa) and Navajo/White mountain (North America). Rectangles display endpoints of confidence intervals for the prevalence of colonization in each of the calendar months shown under the climate characteristics. Point estimates of the percentage of infants colonized in each month is shown by the number within the green rectangle. The climate curves visualize the minimum monthly temperature (in Celsius) in orange and average rainfall (in millimeters) in blue.

We consider a cohort of 223 infants from the Mae La refugee camp located in the proximity of the Thailand-Myanmar border. Nasopharyngeal swabs were collected each month in 2007–2010, starting from the birth until the second birthday of an infant. The colonizing pneumococcal strains were identified using standard protocols^8^,^9^. The prevalence of colonization in the infants varies between the study months (Fig. 1), being highest between February–April, and lowest between August–November in each year. For instance, in 2008 the estimated prevalence is highest in April (84%) and lowest in October (60%), and similarly in 2009 highest in April (87%) and lowest in August (67%). Towards the start and the end of the cohort the sample sizes are smaller and consequently there is more uncertainty about the level of prevalence. The estimated prevalence of colonization appears to change smoothly between the months which could be an indication of correlated seasonal effects. There was some variation in the number of births per calendar month in the cohort, visualized in the Supplementary Figures 5 and 6. This could in principle influence the population dynamics of the pneumococcus carriage. However, as the size of the total population is 40000 individuals and the number of newborns per year is 1500, the observed monthly differences in the numbers of births in our cohort are likely to have a minor impact on the overall dynamics.

The measured monthly rainfall and minimum temperature in the region are also shown for same the study months (Fig. 1). The climatic conditions in Mae La exhibit considerable variation throughout the year; November–March period being the time of dry and cool weather, while May–October corresponds to a warm and humid period. For comparison, we show also (Fig. 1) the prevalence estimates by calendar month from a study on pneumococcal colonization in Kilifi district in Kenya, performed in years 2006–2008^5^, together with the average weather characteristics for the corresponding months. Similarly, we show (Fig. 1) the prevalence estimates from years 1998–2012 and weather summaries from the South-Western United States^6^. In these two examples, observations from several years are aggregated and only the average monthly prevalences are shown, as well as the average weather characteristics. While averaging over several years could distort some signal of the seasonal variation in both the prevalence and weather, all three locations display a pattern where the prevalence of colonization is highest during the months with less rainfall and cooler temperatures.

### Inference about seasonal effects

Regardless of the actual biological mechanisms, a season could ultimately have an impact on either the length of a colonization episode, the transmissibility, or both. The main aim of our analysis is to quantify the possible effects of the seasons on these two quantities. To do this, we partition the study months into seasons using various criteria, and then infer the season-specific acquisition- and clearance rates. The inference is done by fitting a seasonal colonization model to the time-series consisting of monthly observations from each infant in the cohort.

**Figure 2:**
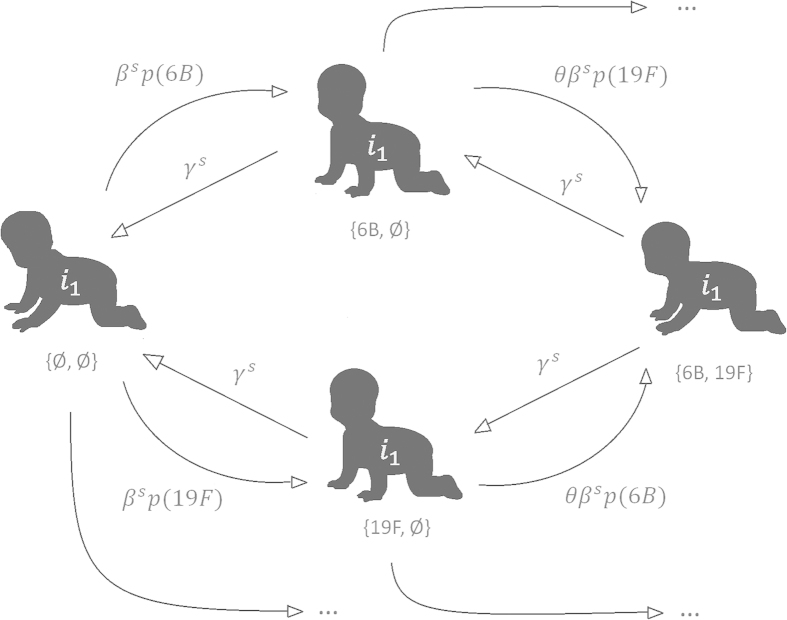
Illustration of the neutral colonization model, where the two example strains represent the serotypes 6B and 19F, respectively. An individual is always considered to be either susceptible (leftmost state), colonized by one strain (middle) or colonized by two strains (rightmost). Arrows from left to right depict acquisitions, and the arrows from right to left correspond to clearance events. Next to each arrow, the corresponding per-day transition probability is shown. Parameter θ represents the competition between the serotypes and p(•) corresponds to the serotype frequency distribution. Transition probabilities representing colonizations with the other strains are constructed in the same manner (states not shown). In the biologically realistic model the transmission- and clearance rates are multiplied with the effects of colonization history of the individual *i*_1_ until the current time and the clearance rates are multiplied with the effect of the serotype that is about to be cleared.

We consider two colonization models with dynamics defined according to the current knowledge about pneumococcal colonization processes. Distinctly from the standard modeling assumptions, we consider the hypothesis that the rate of acquiring an infection (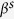) and the rate of clearing an infection (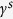) depend on the ongoing season. The first model considered is a so called *neutral model*, the dynamics of which are explained in Fig. 2. The second, a more elaborate model, is defined by introducing an acquired immunity effect and by using previously obtained estimates of serotypic differences in terms of clearance rates. The latter model is subsequently referred to as a *biologically detailed model*. The parameters that govern the additional features in the biologically detailed model were given values based on previous studies. However, since some uncertainty about the exact values of these parameters still remains, the neutral model serves a particular purpose to identify which conclusions regarding seasonal effects remain robust to the modeling assumptions. As a further validation of our results, we also infer the seasonal acquisition rates for newborn individuals that had never been colonized before and the seasonal clearance rates during the first colonization epoch experienced by each newborn. Similarly as in biologically detailed model, we also separately infer the clearance rates during the first colonization, taking into account the strain effects on the rate of clearance. Considering only the first colonization epochs reduces the statistical power, but is helpful for excluding the potentially confounding effects of previous exposures.

Finally, since the prevalence was observed to vary between the seasons, the seasonal differences in the acquisition rate 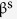
could alternatively be explained by the seasonally changing size of the infectious population. The seasonal changes in the size of infectious population could, in turn, be due to e.g. seasonal variation in the numbers of infants born each month (see Supplementary Chapter 5 for monthly birth rates). To investigate whether the actual *infectivity* of the bacteria varies between the seasons, we consider the ratio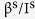, where 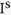 denotes for the prevalence of colonization in the ongoing season.

### Dynamics per calendar month

**Figure 3:**
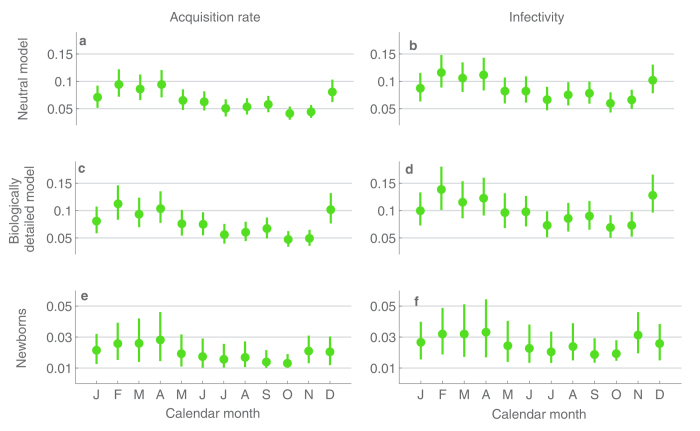
Estimates and 95%-confidence intervals for the calendar month-specific per-day probabilities of acquisition (first column) and infectivity (second column), under the different inference scenarios (rows). In the second column the acquisition rate per individual is scaled by the unknown prevalence of colonization during the ongoing month.

The monthly estimates of acquisition rate and infectivity are visualized in Fig. 3 (exact values of the estimates and their 95% confidence intervals are given in the Supplementary Table 1). Under the neutral model, the estimated acquisition rate per individual
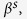
attains its highest value; around 0.09 per day during February–April. The lowest values, approximately 0.04 per day are found in September–October. Moreover, the confidence intervals in these groups of months have no overlap (Fig. 3a). This observation is not explained away by higher prevalence of colonization in these months, as also the estimated infectivity in February (0.11 per day) is nearly twice as high as the infectivity in September (0.06 per day). Similarly, the confidence intervals for the three months of high infectivity (Feb–Apr) have little or no overlap with the confidence intervals for the months of low infectivity (Fig. 3b).

A similar pattern is detected when considering the biologically detailed model (Fig. 3c,d). The same calendar months appear with the correspondingly high/low acquisition rates and the confidence intervals for these months remain non-overlapping. For the two models, the transition in the estimated levels between the early and late months of the year appear smooth and continuous in time, as intermediate values for 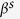
were obtained between the two seasons. When considering the first colonization events of newborns, the confidence intervals for 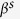
are most clearly concentrated on the low values for September and October (Fig. 3e); the same holding for infectivity (Fig. 3f). The results for newborns are hence congruent with the previously mentioned findings.

In terms of weather, the above mentioned sequences of months correspond to junctions between the different seasons. In all of the three years considered (Fig. 1b), February–April correspond to months when the weather changes from cool and dry into hot and wet, while September–October correspond to the last months of the hot and rainy season. This observation leads us to hypothesize that either the effect of climate on the infectivity comes with a delay, or alternatively it is cumulative in time.

**Figure 4:**
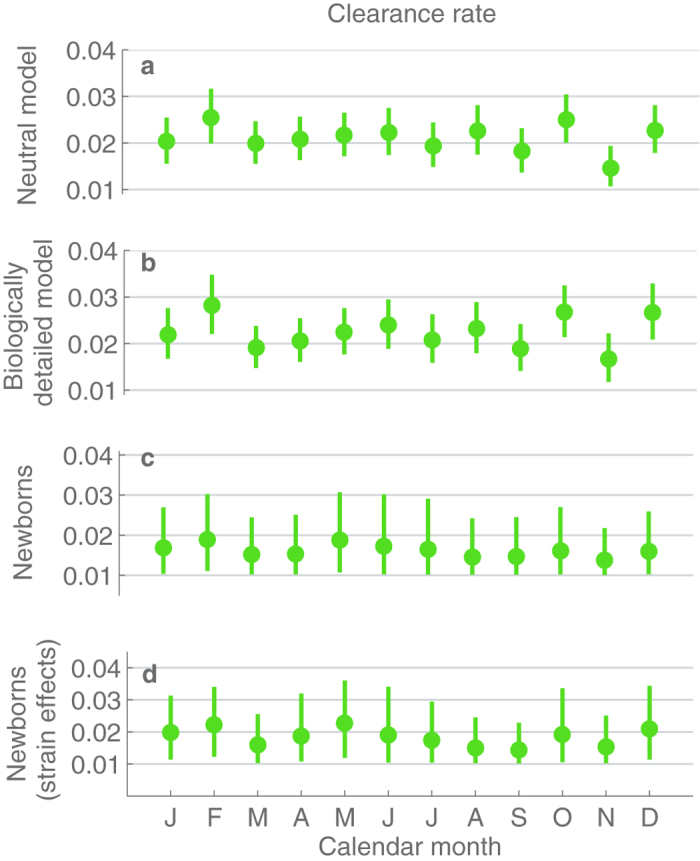
Estimates and 95%-confidence intervals for the calendar month-specific per-day probabilities clearing infection colonization episode, under the different considered inference scenarios (**a–d**).

The estimates for the clearance rate suggest no seasonal pattern, since the monthly values are very similar and there is a substantial overlap between the confidence intervals (Fig. 4). As an exception, the clearance rate in November appears lower, while the estimate for February is slightly higher than the average over the different months. The difference between these two outlying months was mitigated under the biologically detailed model (Fig. 4b). When examining the clearance rate during the first colonization epoch of an infant (Fig. 4c), the two months do not stand out, not even when the clearance rates are adjusted based on the serotype under consideration (Fig. 4d).

### Weather-based dynamics

**Figure 5:**
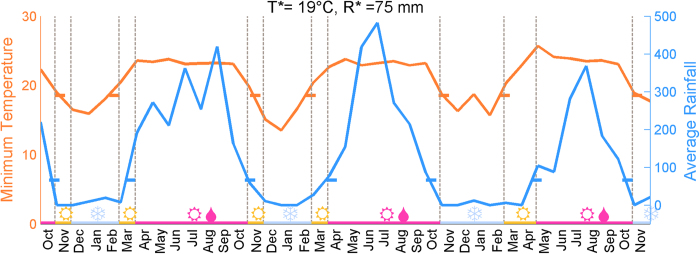
Climate characteristics in the Mae Sot region during the study period and the corresponding season partition for threshold values T* = 19 and R* = 75 mm. On the horizontal axis the study months are shown, and above each month the color indicates the season the month is assigned to. The seasons are hot 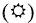, hot and wet 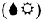 and cool 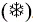. The vertical lines show when the seasons change, while the horizontal ticks intersecting with the temperature and rainfall curves show when the corresponding threshold values are exceeded.

To consider climate-wise colonization dynamics, the sampling months were partitioned into (at most) 4 seasons based on two threshold values: T* for the minimum temperature of the month and R* for the average rainfall of the month. Denoting the minimum temperature and the average rainfall of a month *m* by *T*(*m*) and *R*(*m*), respectively, each month *m* can be assigned to one of the four seasons (Table 1). An example of such a division is shown in Fig. 5. In total, to make robust conclusions we considered six different partitions, using threshold values 19 C or 23 C for the minimum monthly temperature, and 10 mm, 75 mm or 200 mm for the average rainfall. The corresponding climate partitions are visualized in Section 2 of the Supplementary materials, and the results considering the colonization dynamics are summarized in the Supplementary Table 2. The partitions could have also been determined for instance based on the mean monthly temperature. However, in the study population the mean and minimum temperature were highly correlated, as shown in Supplementary Figure 7, and therefore the partitions based on the two variables would be similar. Also, the exact threshold values used are to an extent arbitrary, and therefore we studied the robustness of our findings across several possible choices of thresholds.

**Figure 6:**
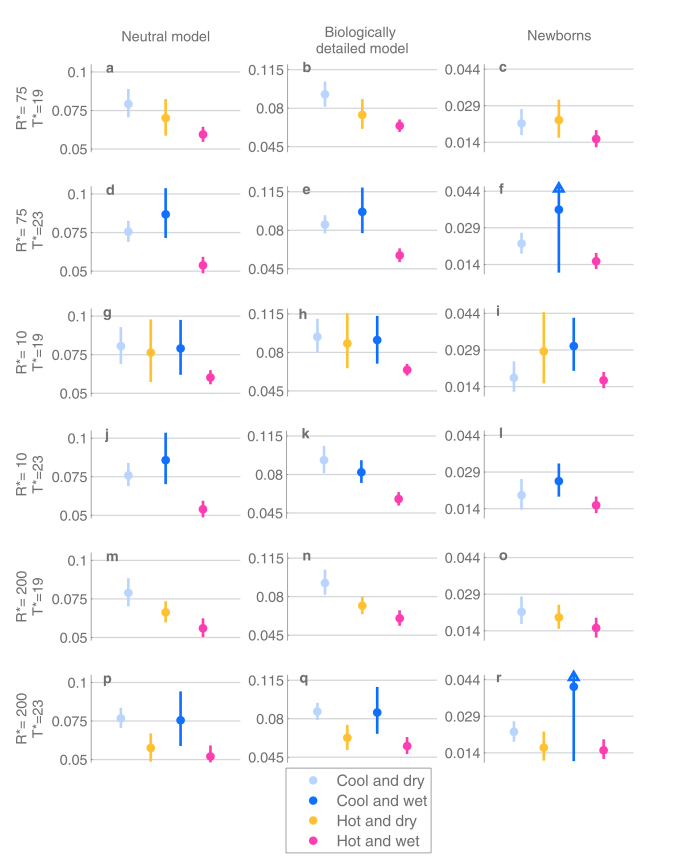
The estimates and 95%-confidence intervals for the acquisition rates per individual per day under different inference scenarios (columns) and season thresholds (rows). Each row corresponds to a specific choice of thresholds for the minimum temperature and average rainfall, an example of which was shown in detail in Fig. 5. These choices define partitions of the study months into the four seasons shown by markers at the bottom of the figure, such that the estimates and confidence intervals are colored according to the corresponding season. For the results concerning ‘newborns’ a triangle symbol indicates when a confidence interval is not fully visualized due to visual clarity of the whole figure.

**Figure 7:**
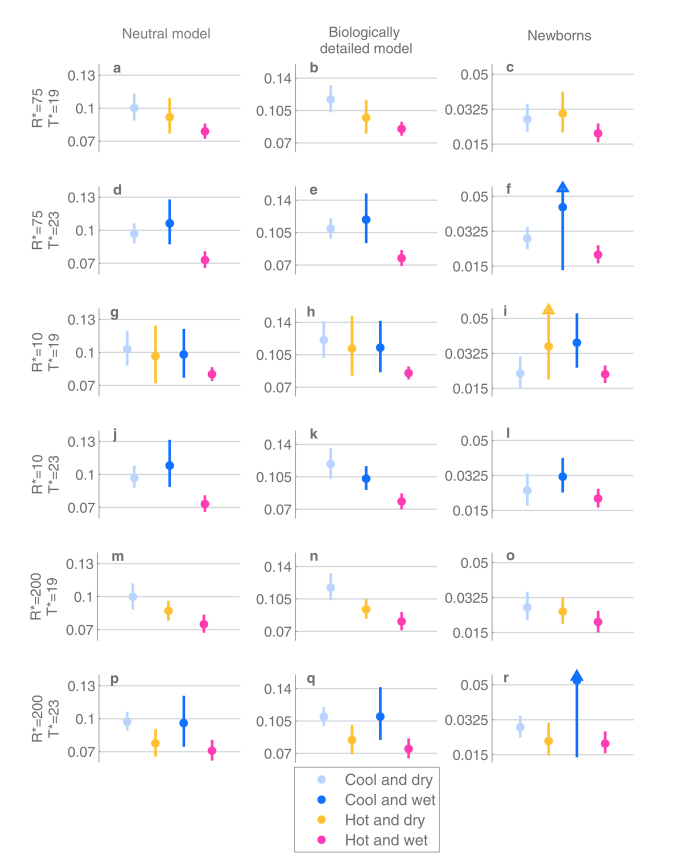
The estimates and 95%-confidence intervals for the infectivity per individual per day under different scenarios of inference (columns) and season-threshold values (rows). All figure elements are defined as in Fig. 6.

The most important conclusion from the climate-based analysis is that under all the different season partitions and both the neutral and the biologically detailed model the acquisition rate and the infectivity are lowest for the hot and wet season, and that the confidence intervals for 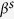 never overlap between the hot/wet season and the cool/dry season (Figs 6 and 7). As an example, when T* = 19 C and R* = 75 mm were used as thresholds, the point estimate for the infectivity in cool and dry season was 0.1169 per day, with a confidence interval (0.103, 0.1325), and in hot and wet season the corresponding point estimate was 0.0850, with confidence interval (0.0779, 0.0927). When considering the newborns and their first colonization epoch, the trends in infectivity and acquisition rates are similar, but the differences between the seasons are not always statistically significant due to the much smaller amount of data available.

**Figure 8:**
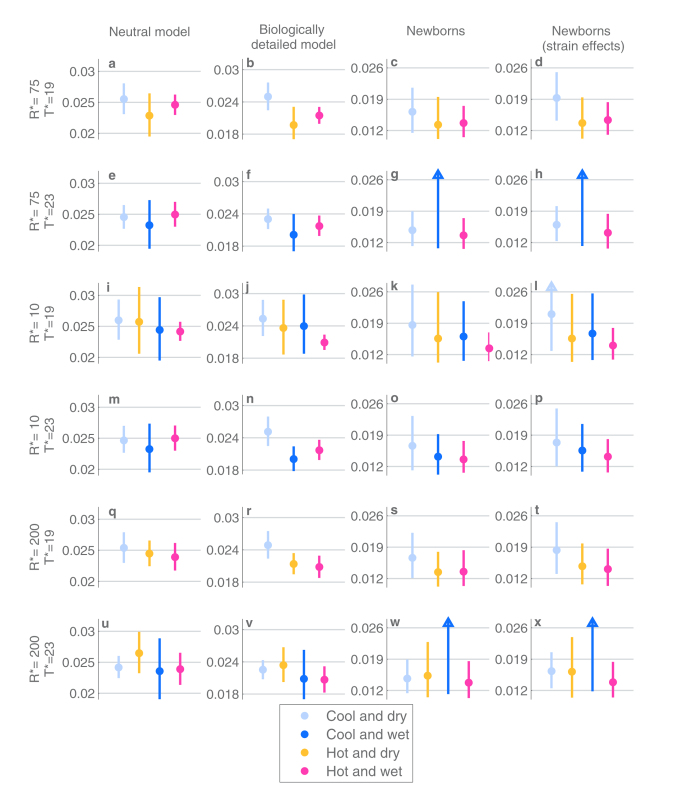
The estimates and 95%-confidence intervals for the clearance rates per individual per day under different inference scenarios (columns) and different season-thresholds (rows). All figure elements are defined as in Fig. 6.

The climate-based analysis for the clearance rate yields very similar results to the calendar month –based analysis (Fig. 8). There is no clear indication of the clearance rate alternating between the seasons, as the confidence intervals for the estimates between the seasons overlap substantially. While the clearance rate appears slightly higher in the cool and dry season, there is otherwise no systematic ordering of the seasons with respect to their rates of clearance.

## Discussion

Our combined results suggest that if the weather conditions represent a main determinant of the seasonal dynamics of the pneumococcus, then these bacteria are less infectious when it is hot and rainy season, while being optimally transmitted during the coolest and driest months. We additionally conclude that the climate or the time of the year in general seems not to have a significant effect on the rate of clearance.

When calendar months were analyzed separately, we identified months of high and low transmissibility, residing at the end of the cool/dry season and the hot/wet season, respectively. This led us to hypothesize that the effect of climate on infectivity is cumulative or comes with a delay. We inferred that in monthly terms the clearance rate remains surprisingly constant, albeit that it appeared slightly lower than on average in October. This could be explained for instance by bacterial-viral interactions. Namely, it has been previously detected that October is the month of the peak prevalence of respiratory syncytial virus (RSV) in our study population^10^,^11^. Previous studies have shown that by binding to the pneumococcal surface proteins, RSV enhances both the adherence to the human epithelium and the expression of pneumococcal virulence genes^12^. Altogether, this results in longer stay in the nasopharynx^13^. This is in harmony with our finding according to which the clearance rate is lowest at the time of highest RSV prevalence.

There several mechanisms of interaction between the respiratory pathogens and several known interactions between them^14^. These different interactions could possibly explain seasonality in pneumococcal carriage even in a greater detail. For instance, it appears that the first months of the year represent the time of peak incidence of Adenovirus^10^. The interactions between the pneumococcus and Adenovirus are not well known, yet one study^15^ found a negative association between the colonization of the two species, while another study^16^ suggested that Adenovirus is also capable of enhancing the adherence of particular pneumococcal strains to the epithelial cells.

It is known that for instance influenza transmission is influenced largely by humidity and temperature^17^ . Recently, it was shown that nose temperature modifies innate responses against rhinovirus causing the common cold, such that the virus can replicate better at cooler temperatures^18^. It is reasonable to hypothesize that similar mechanisms could explain the effects of climate for bacteria too. Dry and cold air could both influence the conditions of the nasopharyngeal mucosa, and also the infectious droplets and the survival of bacteria in them. Some hint of distinguishing between the possible mechanisms was given by the observation in a recent colonization study^6^, which found that during the months of higher prevalence, colonized individuals were also on average colonized with higher densities of bacteria. If this observation can be generalized to our results, then a larger colony of bacteria seems not to induce a longer colonization time.

To our knowledge this is a first thorough statistical analysis on the actual colonization dynamics underlying the seasonal patterns in the prevalence of colonization of the pneumococcus. In the analysis we have taken several precautions to ensure that the results are not explained away by confounding factors. For instance, the separate analysis of the newborns and their first colonizations was motivated by the uncertainty about the exact effects of the acquired immunity caused by past colonizations. On the other hand, the biologically detailed model was constructed to utilize most of the current understanding about the acquired immunity and the colonization dynamics of different pneumococcal serotypes in general. We considered also the neutral model to examine whether the results were too sensitive to these biological details, the conclusion being that they were robust. It is worth noticing though that we are not modelling the daily fluctuations in the weather within any particular month, but assume that eventual effects are based on the longer-term conditions.

We emphasize that the data analyzed in this study should be ideal for the purpose of examining effects of the climatic conditions. First, as the study population is large enough, the effects of small random ‘microepidemics’ are not expected to dominate over the possible non-random seasonal effects. Second, the data were collected monthly, regardless of the disease status, and thus the possible seasonality in pneumococcal disease should not affect our results. Last, the cohort consisted of young infants, who should be minimally influenced by seasonal behavioral patterns of humans. On the contrary, colonization studies for instance from day care center attendees might be heavily influenced by holidays, as was noticed in a study on Swedish children between ages 0–7^19^, in which Christmas, Easter and summer holidays had a significant effect on the prevalence of colonization. Nevertheless, as always when studying infectious diseases of humans, it is difficult to disentangle effects of the host and pathogen behavior. The infants studied here could for instance have been influenced by a seasonal behavior of their mothers. We gain confidence in that this is not the case by notifying the seasonality of the prevalence of colonization in South-East Asia, Africa and North America shown in Fig. 1. Also there the relationship between the climate and the prevalence of colonization is somewhat similar to the data we have analyzed. Since the societies and holiday seasons would be very likely different, it is reasonable to claim, although more detailed analysis is needed for confirmation, that the climate does have an effect, which acts similarly across the different geographical regions.

## Methods

### Data

Data consist of nasopharyngeal swabs collected from 223 children as reported in an earlier genomics study^9^. Here we only considered the individuals from whom all the collected swabs were cultured. As part of the study described by Turner *et al.*^8^, between October 2007 and November 2008, all pregnant women attending the Shoklo Malaria Research Unit (SMRU) antenatal clinic at 28–30 weeks gestation were invited to consent to their infant’s participation in a pneumonia cohort study approved by the Centre for Tropical Medicine, University of Oxford, Oxford, United Kingdom. Written informed consent was obtained from the mothers prior to study enrolment. Ethical approval was granted by the ethics committees of the Faculty of Tropical Medicine, Mahidol University, Thailand (MUTM-2009-306) and Oxford University, UK (OXTREC-031-06). All analyses and methods were carried out in accordance with the approved guidelines. Nasopharyngeal swabs were collected according to the WHO pneumococcal colonization detection protocol^20^. The follow-up was monthly, terminating at the age of 24 months. Nasopharyngeal swabs were collected and processed according to the standard WHO pneumococcal detection protocol^21^. All pneumococcal isolates were serotyped in-house by latex agglutination (using the method developed by the MRC laboratories in Gambia), with Quellung to confirm equivocal results. The weather data for the Mae Sot region was obtained from Mae Sot Meteorological Station, Thai Meteorological Department, and for Kilifi and Navajo from worldweatheronline.com.

### Models for colonization

#### Neutral colonization model

The model for colonization dynamics considers the colonization process of each single individual, assuming common parameters governing the probabilities of state transitions for all infants. Each individual is assumed to be colonized by at most two distinct strains (serotypes) at any time. We describe the dynamics using a discrete-time Markov model with a time-unit of one day. The unknown model parameters are: the season-specific per-day probability of a susceptible individual to be infected (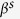) and the season-specific per-day probability of a colonized individual to clear an infection (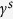). We denote the individual colonization states with the following notation: (Ø, Ø), (x, Ø), (x, y), which correspond to a susceptible individual, an individual colonized with a single strain x, and an individual colonized with two strains x and y, respectively. We use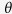 to denote for the relative rate of getting colonized by another strain once already colonized with one pneumococcal strain. We set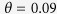 based on two previous studies estimating the relative co-colonization rate under a neutral model^22^. In addition, we denote with 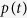 the frequency of strain *t* in the overall population serotype distribution. Then, conditional on the current season being *s*, the neutral colonization model is defined by the one-day transition probabilities given in Table 2 for all possible combinations of strains x and y.

Under the neutral model we assume that the clearance rate of a strain is affected by the season only, not by the strain identity. The model has a structure of a standard SIS model with co-colonization states, similar to other studies on pneumococcal colonization dynamics^23^,^22^, but here the rates are not time-homogenous as they depend on the season *s* to which a day belongs. Also, the seasonal acquisition rate does not explicitly depend on the number of infectious at the date. Instead, we assume that conditional on the seasonal infection hazard 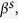 the individual time-series are independent of each other. The frequency distribution for the serotypes *p*(*x*), which sets the probability to acquire a particular strain, was obtained from the data using the empirical distribution of all serotypes in the collected samples.

#### Biologically detailed model

The biologically detailed model is obtained by modifying the rates of the neutral model in Table 2. The season-specific clearance rates are multiplied by a serotype-specific effect, accounting for the fact that different serotypes are known to persist in the nasopharynx for different lengths of time. We give values for the serotype-specific effects based on a previous extensive study on the clearance rates^24^. The exact details on specifying the serotype effects for the clearance rates are explained in the Supplementary materials Section 3.2.

To model the effects of acquired immunity as well, the seasonal rates are adjusted based on the previous exposures of the individual at question. There is evidence that past colonization with a serotype can yield partial protection for acquisition of the same serotype later^25^. Recent studies have concluded that there exists a serotype-independent acquired immunity, which manifests itself in increasing the clearance rate in the future colonizations for all serotypes. Based on this previous knowledge, we parameterize the biologically detailed model to have both of these features, assuming that the effects are multiplicative on the rates defined in Table 2. In summary, we assume that the relative rate of acquisition of a serotype, once already having been colonized with it earlier is 0.7, and that the relative rate of clearing any serotype, after having been colonized with an arbitrary serotype is 1.25. We do not consider immunity derived from the mother, e.g. through breastfeeding. This is because a previous study^26^ concluded that the concentrations of the maternally derived antibodies in the children during the first two years of life were below a level required to influence the colonization outcomes. Moreover, the increases in antibody concentrations were mostly observed to be associated with previous colonizations, rather than being obtained from the mother. Supplementary material Sections 3.2 and 3.3 provide all the quantitative characteristics of the biologically detailed model.

### Statistical inference

#### Likelihood calculations

Denote by 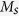 the one-day transition matrix in season *s*, which contains the transition probabilities as defined by the colonization model (either as in Table 2 or in the Supplementary material). Denote with 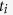
the vector of sampling times for the individual *i,* and with 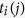 the *j*’th sampling time of individual *i*. Further, denoting with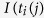) the colonization status of the individual *i* at time 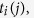
the transition matrix for the sampling interval
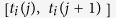
is given by:


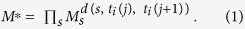


Above *s* indexes the seasons in the order of appearance, starting from the season in 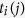 to 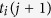. In the matrix exponent,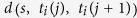 denotes the number of days between the sampling days 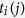 and 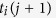
that belong to season 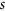. The likelihood of transition 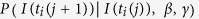
is given by the element of matrix M*, that corresponds to the observations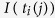 and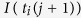. The likelihood contribution of individual *i* is then:


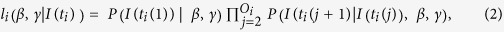


where 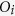 is the number of observations from individual *i*. As individuals are known to be born as susceptible, for the likelihood of first observation it always holds that 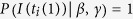. Full likelihood of the data equals:


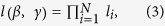


where *N* is the number of individuals.

#### Inference about colonization model parameters

We conducted Bayesian inference on the parameters of interest, which allowed us to investigate the uncertainty related to the statistical results in light of the data and all that is known beforehand. Notice that in the Results section of the article we refer to the simulation-based 95% credible intervals as ‘confidence intervals’ to follow established statistical nomenclature familiar to most readers.

Model fitting was done separately for the different possible partitions of the cohort months in the seasons and for the three different inference scenarios. Posterior samples were obtained using a Metropolis-Hastings Markov chain Monte Carlo (MCMC) sampling- technique. We simulated 20 MCMC-chains initiated at different starting points until convergence, with the initial samples discarded. To ensure the convergence, we computed the interval-based Gelman-Rubin statistic^27^ for the chains and continued sampling the chain until this statistic was below 1.1. Examples of trace plots of the MCMC-runs are provided in supplementary chapter 4.4. For detailed information about the prior distributions used and the implementation of the MCMC simulations, see the section 4 in the Supplementary materials.

The inference procedure considering the newborns and their first colonization epochs is described in the Supplementary materials chapter 4.5.

#### Estimation of prevalence 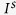 and infectivity 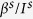


As seen from the first panel of Fig. 1, the prevalence of colonization varies between the seasons. Thus in principle the inferred variation in acquisition rate 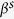 could be due to varying size of the infectious population. To rule this out and to estimate the actual infectivity of the infectious population, we estimate the unknown prevalence of colonization for every season, and then factor it out from the estimate of 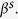 This is accomplished by assuming that for every a season *s*, there exists a parameter 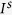 describing the prevalence of colonization in that season. We assume that the likelihood of 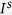 corresponds to the binomial distribution:


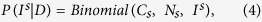


where 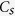 is the number of infants observed to be colonized during season *s* and 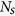 is the number of samples collected in season *s*. We assume 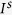 has uniform prior distribution on interval (0, 1) as the prior distribution, which results in the posterior distribution being a 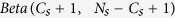.

Finally, the posterior distribution for the infectivity, i.e. the ratio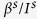, is obtained by integrating over the uncertainty related to the prevalence and the uncertainty related to the acquisition rate for a given season:


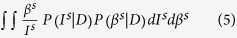


The above integral can be approximated with a Monte Carlo integration procedure, once the two posterior distributions are obtained. We approximated the distribution of 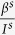 by sampling 5000 realizations from 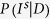 and from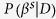.

## Additional Information

**How to cite this article**: Numminen, E. *et al.* Climate induces seasonality in pneumococcal transmission. *Sci. Rep.* **5**, 11344; doi: 10.1038/srep11344 (2015).

## Supplementary Material

Supplementary Information

## Figures and Tables

**Table 1:** Partition of the cohort study period according to climatic conditions.

	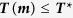	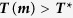
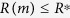	cool and dry	hot and dry
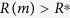	cool and wet	hot and wet

**Table 2:** Transition rates between different colonization states under the neutral model. Labels x and y correspond to any two strains (serotypes).

**current state**	**next state**	**probability**
(Ø, Ø)	(x, Ø)	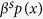
(x, Ø)	(Ø, Ø)	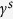
(x, Ø)	(x, y)	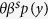
(x, y)	(x, Ø)	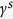

**Figure:**
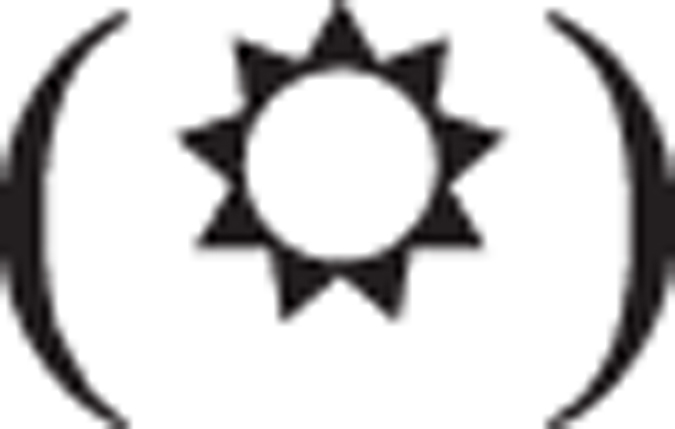


**Figure:**
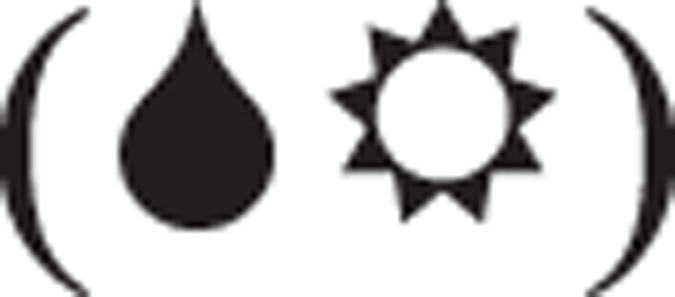


**Figure:**