# Corrigendum: Climate induces seasonality in pneumococcal transmission

**DOI:** 10.1038/srep23307

**Published:** 2016-03-29

**Authors:** Elina Numminen, Claire Chewapreecha, Claudia Turner, David Goldblatt, Francois Nosten, Stephen D. Bentley, Paul Turner, Jukka Corander

Scientific Reports 5: Article number: 11344;10.1038/srep11344 published online: 06122015; updated: 03292016

The Acknowledgements section in this Article is incomplete.

“We thank Ben Cooper for helpful discussions, Ritva Syrjänen for pointing out useful references on the seasonality of colonization, Marc Lipsitch for advice in parametrizing the effects of previous exposures in the model and Dan Weinberger on sharing the point estimates on the Navajo colonization study, visualized in Fig. 1. E.N. and J.C. were funded by ERC grant no. 239784 and Academy of Finland grant no. 251170. P.T. was funded by the Wellcome Trust of Great Britain (Grant No. 083735/Z/07/Z).”

should read:

“We thank Ben Cooper for helpful discussions, Ritva Syrjänen for pointing out useful references on the seasonality of colonization, Marc Lipsitch for advice in parametrizing the effects of previous exposures in the model and Dan Weinberger on sharing the point estimates on the Navajo colonization study, visualized in Fig. 1. E.N. and J.C. were funded by ERC grant no. 239784 and Academy of Finland grant no. 251170. P.T. was funded by the Wellcome Trust of Great Britain (Grant No. 083735/Z/07/Z). This research was supported by the National Institute for Health Research Biomedical Research Centre at Great Ormond Street Hospital for Children NHS Foundation Trust and University College London.”

