# Supplementary material for: Climate induces seasonality in pneumococcal transmission
Source: Sci Rep. 2015 Jun 12;5:11344. doi: 10.1038/srep11344 (PMC4464306; doi:10.1038/srep11344)
Supplement: Supplementary Information [file srep11344-s1.pdf]

Supplementary materials for the article entitled: *'Climate induces seasonality in pneumococcal transmission'*

Elina Numminen, Claire Chewapreecha, Claudia Turner, David Goldblatt, Francois Nosten, Stephen D. Bentley, Paul Turner, Jukka Corander

Contents

1 Results 2
1.1 Calendar months as seasons 2
1.2 Seasons based on climate 3
2 Climate seasons used in the modeling 4
3 Model specifications 7
3.1 The reduced state space of the Markov model for a particular sampling interval 7
3.2 Strain-effects in the biologically detailed model 9
3.3 Effects of exposures in the biologically detailed model 10
3.4 Sampling errors 12
4 Inference 13
4.1 The prior distributions 13
4.2 The proposal distribution in the MCMC sampler 14
4.3 Details of the MCMC runs 14
4.4 Trace plots 16
4.5 Inference considering only the first colonization event of the newborns 17
5 Monthly birth rates in the cohort 18
6 The minimum-, mean and maximal temperatures during the study 20
7 Supplementary References 21

1 Results

1.1 Calendar months as seasons

In Supplementary Table 1 below we show the posterior summaries for clearance rate γ, acquisition rate β and the infectivity, denoted with $\frac{\beta}{I}$, when months were considered as seasons.

	Neutral model			Biologically detailed model			Newborns			
Parameter	γ	β	$\frac{\beta}{I}$	γ	β	$\frac{\beta}{I}$	γ	γ (strain effects)	β	$\frac{\beta}{I}$
January	0.0203<br>0.0156<br>0.0254	0.0708<br>0.0518<br>0.0922	0.0874<br>0.0635<br>0.1153	0.0219<br>0.0168<br>0.0276	0.0806<br>0.0587<br>0.1073	0.0998<br>0.0729<br>0.1331	0.0169<br>0.0104<br>0.0270	0.0198<br>0.0114<br>0.0313	0.0214<br>0.0127<br>0.0320	0.0266<br>0.0155<br>0.0398
February	0.0255 ↑<br>0.0199<br>0.0316	0.0944 ↑<br>0.0720<br>0.1221	0.1161 ↑<br>0.0888<br>0.1479	0.0282 ↑<br>0.0221<br>0.0348	0.1122↑<br>0.0833<br>0.1461	0.1386↑<br>0.1012<br>0.1804	0.0189↑<br>0.0111<br>0.0303	0.0222<br>0.0122<br>0.0340	0.0258↑<br>0.0152<br>0.0392	0.0319↑<br>0.0188<br>0.0487
March	0.0199<br>0.0155<br>0.0246	0.0861<br>0.0657<br>0.1124	0.1058<br>0.0807<br>0.1347	0.0191<br>0.0148<br>0.0238	0.0934<br>0.0696<br>0.1235	0.1150<br>0.0861<br>0.1538	0.0152<br>0.0103<br>0.0245	0.0159<br>0.0103<br>0.0255	0.0260<br>0.0140<br>0.0419	0.0319 ↑<br>0.0172<br>0.0511
April	0.0208<br>0.0163<br>0.0256	0.0941<br>0.0708<br>0.1207	0.1116<br>0.0834<br>0.1431	0.0206<br>0.0161<br>0.0254	0.1036<br>0.0775<br>0.1352	0.1226<br>0.0911<br>0.1601	0.0153<br>0.0103<br>0.0251	0.0187<br>0.0108<br>0.0320	0.0281<br>0.0144<br>0.0461	0.0331<br>0.0169<br>0.0543
May	0.0216<br>0.0171<br>0.0265	0.0653<br>0.0478<br>0.0855	0.0822<br>0.0596<br>0.1070	0.0224<br>0.0177<br>0.0276	0.0759<br>0.0543<br>0.1014	0.0963<br>0.0682<br>0.1317	0.0188<br>0.0107<br>0.0307	0.0227 ↑<br>0.0119<br>0.0360	0.0192<br>0.0110<br>0.0317	0.0244<br>0.0139<br>0.0404
June	0.0223<br>0.0174<br>0.0275	0.0625<br>0.0467<br>0.0818	0.0823<br>0.0614<br>0.1090	0.0240<br>0.0189<br>0.0294	0.0748<br>0.0548<br>0.0966	0.0979<br>0.0712<br>0.1267	0.0173<br>0.0103<br>0.0302	0.0190<br>0.0105<br>0.0341	0.0173<br>0.0102<br>0.0290	0.0226<br>0.0133<br>0.0380
July	0.0194<br>0.0148<br>0.0244	0.0504<br>0.0359<br>0.0673	0.0663<br>0.0470<br>0.0901	0.0208<br>0.0159<br>0.0263	0.0560<br>0.0396<br>0.0756	0.0733<br>0.0513<br>0.0987	0.0165<br>0.0102<br>0.0291	0.0174<br>0.0105<br>0.0294	0.0156<br>0.0103<br>0.0256	0.0205<br>0.0133<br>0.0334
August	0.0226<br>0.0175<br>0.0281	0.0533<br>0.0396<br>0.0691	0.0753<br>0.0560<br>0.0986	0.0232<br>0.0180<br>0.0289	0.0605<br>0.0441<br>0.0796	0.0858<br>0.0619<br>0.1141	0.0145<br>0.0102<br>0.0242	0.0150<br>0.0102<br>0.0245	0.0168<br>0.0106<br>0.0271	0.0238<br>0.0149<br>0.0389
September	0.0182<br>0.0136<br>0.0231	0.0579<br>0.0437<br>0.0735	0.0783<br>0.0592<br>0.0993	0.0189<br>0.0141<br>0.0242	0.0669<br>0.0490<br>0.0872	0.0900<br>0.0651<br>0.1173	0.0147<br>0.0102<br>0.0245	0.0143 ↓<br>0.0101<br>0.0229	0.0140<br>0.0101<br>0.0214	0.0188 ↓<br>0.0135<br>0.0292
October	0.0250<br>0.0200<br>0.0304	0.0411↓<br>0.0300<br>0.0539	0.0599↓<br>0.0434<br>0.0798	0.0268<br>0.0214<br>0.0325	0.0472 ↓<br>0.0343<br>0.0624	0.0689 ↓<br>0.0501<br>0.0916	0.0161<br>0.0103<br>0.0270	0.0192<br>0.0106<br>0.0336	0.0131 ↓<br>0.0101<br>0.0190	0.0192<br>0.0147<br>0.0279
November	0.0146↓<br>0.0107<br>0.0194	0.0444<br>0.0333<br>0.0566	0.0660<br>0.0498<br>0.0845	0.0167 ↓<br>0.0118<br>0.0222	0.0493<br>0.0356<br>0.0650	0.0732<br>0.0524<br>0.0978	0.0138 ↓<br>0.0101<br>0.0217	0.0154<br>0.0102<br>0.0251	0.0210<br>0.0131<br>0.0309	0.0313<br>0.0194<br>0.0460
December	0.0227<br>0.0178<br>0.0281	0.0807<br>0.0623<br>0.1032	0.1020<br>0.0783<br>0.1307	0.0266<br>0.0209<br>0.0329	0.1016<br>0.0765<br>0.1319	0.1280<br>0.0965<br>0.1658	0.0160<br>0.0104<br>0.0259	0.0210<br>0.0114<br>0.0343	0.0204<br>0.0119<br>0.0304	0.0258<br>0.0149<br>0.0384

Supplementary Table 1: The posterior mean estimates and the 95% confidence intervals for the month-specific parameters under the three scenarios considered. In each cell, the first number is the posterior mean, and the next two numbers denote the upper and lower limits of the confidence intervals, respectively. We use symbols ↓ and ↑ to denote for the minimum and maximum values of the posterior means in each column.

1.2 Seasons based on climate

In Supplementary table 2 we show the posterior summaries for clearance rate γ, acquisition rate β and the infectivity, denoted with $\frac{\beta}{I}$, under different partitions of study months into climate seasons.

Model / Parameter / Season		Neutral model			Biologically detailed model			Newborns			
		γ	β	$\frac{\beta}{I}$	γ	β	$\frac{\beta}{I}$	γ	γ (strain effects)	β	$\frac{\beta}{I}$
T*=19<br>R*=75	cool & dry	0.0255<br>0.0231<br>0.0280	0.0792<br>0.0705<br>0.0889	0.1003 ↑<br>0.0889<br>0.1131	0.0250↑<br>0.0224<br>0.0276	0.0925<br>0.0814<br>0.1042	0.1169 ↑<br>0.1030<br>0.1325	0.0162<br>0.0115<br>0.0216	0.0193<br>0.0142<br>0.0250	0.0217<br>0.0170<br>0.0276	0.0275<br>0.0213<br>0.0351
	hot & dry	0.0228<br>0.0195<br>0.0264	0.0701<br>0.0587<br>0.0824	0.0920<br>0.0769<br>0.1093	0.0197<br>0.0164<br>0.0231	0.0740<br>0.0612<br>0.0882	0.0972<br>0.0800<br>0.1165	0.0133<br>0.0101<br>0.0195	0.0137<br>0.0102<br>0.0194	0.0231<br>0.0159<br>0.0315	0.0303 ↑<br>0.0208<br>0.0413
	hot & wet	0.0255<br>0.0230<br>0.0262	0.0594<br>0.0547<br>0.0644	0.0788 ↓<br>(0.0721<br>0.0860)	0.0215<br>0.0199<br>0.0231	0.0640<br>0.0585<br>0.0698	0.0850↓<br>0.0779<br>0.0927	0.0137<br>0.0105<br>0.0175	0.0143<br>0.0111<br>0.0183	0.0154<br>0.0120<br>0.0190	0.0204 ↓<br>0.0159<br>0.0253
T*=23<br>R*=75	cool & dry	0.0245<br>0.0227<br>0.0265	0.0756<br>0.0690<br>0.0826	0.0969<br>0.0880<br>0.1062	0.0230<br>0.0212<br>0.0250	0.0852<br>0.0773<br>0.0937	0.1092<br>0.0985<br>0.1205	0.0148<br>0.0112<br>0.0190	0.0160<br>0.0123<br>0.0201	0.0226<br>0.0185<br>0.0269	0.0290<br>0.0235<br>0.0347
	cool & wet	0.0232<br>0.0195<br>0.0273	0.0868<br>0.0715<br>0.1038	0.1063 ↑<br>0.0872<br>0.1280	0.0201<br>0.0166<br>0.0240	0.0968<br>0.0775<br>0.1188	0.1189↑<br>0.0936<br>0.1472	0.0324<br>0.0107<br>0.0915	0.0428<br>0.0113<br>0.1193	0.0364<br>0.0107<br>0.1148	0.0445 ↑<br>0.0131<br>0.1404
	hot & wet	0.0250<br>0.0230<br>0.0270	0.0538<br>0.0486<br>0.0593	0.0730 ↓<br>0.0657<br>0.0808	0.0217<br>0.0199<br>0.0237	0.0571<br>0.0512<br>0.0635	0.0774 ↓<br>0.0689<br>0.0861	0.0136<br>0.0106<br>0.0174	0.0142<br>0.0107<br>0.0184	0.0153<br>0.0121<br>0.0187	0.0208 ↓<br>0.0165<br>0.0255
T*=19<br>R*=10	cool & dry	0.0260<br>0.0229<br>0.0293	0.0805<br>0.0690<br>0.0929	0.1029 ↑<br>0.0880<br>0.1198	0.0253<br>0.0221<br>0.0289	0.0944<br>0.0799<br>0.1105	0.1209↑<br>0.1018<br>0.1410	0.0186<br>0.0116<br>0.0276	0.0210<br>0.0128<br>0.0310	0.0176<br>0.0119<br>0.0243	0.0225<br>0.0152<br>0.0311
	hot & dry	0.0257<br>0.0206<br>0.0313	0.0764<br>0.0574<br>0.0979	0.0967<br>0.0720<br>0.1244	0.0236<br>0.0187<br>0.0289	0.0881<br>0.0656<br>0.1157	0.1116<br>0.0826<br>0.1471	0.0156<br>0.0102<br>0.0259	0.0156<br>0.0104<br>0.0255	0.0284<br>0.0153<br>0.0445	0.0360<br>0.0194<br>0.0566
	cool & wet	0.0244<br>0.0195<br>0.0297	0.0791<br>0.0620<br>0.0975	0.0980<br>0.0771<br>0.1214	0.0240<br>0.0188<br>0.0298	0.0913<br>0.0698<br>0.1132	0.1128<br>0.0862<br>0.1416	0.0160<br>0.0106<br>0.0239	0.0167<br>0.0108<br>0.0257	0.0306<br>0.0205<br>0.0422	0.0379 ↑<br>0.0254<br>0.0526
	hot & wet	0.0242<br>0.0227<br>0.0257	0.0603<br>0.0559<br>0.0650	0.0802 ↓<br>0.0740<br>0.0865	0.0209<br>0.0195<br>0.0224	0.0641<br>0.0591<br>0.0696	0.0851↓<br>0.0783<br>0.0924	0.0134<br>0.0105<br>0.0169	0.0140<br>0.0109<br>0.0179	0.0166<br>0.0134<br>0.0200	0.0221 ↓<br>0.0177<br>0.0265
T*=23<br>R*=10	cool & dry	0.0247<br>0.0227<br>0.0270	0.0759<br>0.0690<br>0.0840	0.0970<br>0.0877<br>0.1079	0.0251<br>0.0225<br>0.0280	0.0930<br>0.0812<br>0.1059	0.1188↑<br>0.1030<br>0.1357	0.0166<br>0.0112<br>0.0233	0.0174<br>0.0120<br>0.0249	0.0195<br>0.0135<br>0.0262	0.0249<br>0.0173<br>0.0335
	cool & wet	0.0233<br>0.0195<br>0.0273	0.0857<br>0.0703<br>0.1036	0.1080 ↑<br>0.0887<br>0.1315	0.0201<br>0.0178<br>0.0224	0.0820<br>0.0723<br>0.0928	0.1030<br>0.0908<br>0.1164	0.0142<br>0.0102<br>0.0192	0.0156<br>0.0109<br>0.0215	0.0253<br>0.0190<br>0.0325	0.0319 ↑<br>0.0240<br>0.0413
	hot & wet	0.0250<br>0.0230<br>0.0270	0.0539<br>0.0488<br>0.0594	0.0732 ↓<br>0.0660<br>0.0810	0.0217<br>0.0199<br>0.0236	0.0576<br>0.0517<br>0.0639	0.0783↓<br>0.0699<br>0.0872	0.0136<br>0.0106<br>0.0177	0.0142<br>0.0107<br>0.0181	0.0155<br>0.0122<br>0.0189	0.0210 ↓<br>0.0165<br>0.0258
T*=19<br>R*=200	cool & dry	0.0254<br>0.0230<br>0.0279	0.0789<br>0.0702<br>0.0884	0.0999 ↑<br>0.0886<br>0.1121	0.0249<br>0.0224<br>0.0275	0.0924<br>0.0817<br>0.1046	0.1172 ↑<br>0.1037<br>0.1328	0.0166<br>0.0121<br>0.0222	0.0183<br>0.0130<br>0.0246	0.0218<br>0.0168<br>0.0280	0.0275 ↑<br>0.0212<br>0.0354

	hot & dry	0.0245<br>0.0224<br>0.0266	0.0664<br>0.0598<br>0.0734	0.0870<br>0.0781<br>0.0963	0.0214<br>0.0195<br>0.0234	0.0717<br>0.0641<br>0.0798	0.0938<br>0.0836<br>0.1048	0.0134<br>0.0102<br>0.0180	0.0147<br>0.0107<br>0.0198	0.0195<br>0.0149<br>0.0247	0.0256<br>0.0195<br>0.0326
	hot & wet	0.0239<br>0.0217<br>0.0262	0.0560<br>0.0503<br>0.0623	0.0749 ↓<br>0.0671<br>0.0835	0.0208<br>0.0188<br>0.0229	0.0601<br>0.0535<br>0.0676	0.0806↓<br>0.0712<br>0.0910	0.0135<br>0.0103<br>0.0183	0.0141<br>0.0104<br>0.0187	0.0152<br>0.0113<br>0.0193	0.0203 ↓<br>0.0151<br>0.0259
T*=23<br>R*=200	cool & dry	0.0242<br>0.0224<br>0.0260	0.0767<br>0.0705<br>0.0836	0.0973↑<br>0.0894<br>0.1063	0.0225<br>0.0208<br>0.0243	0.0864<br>0.0788<br>0.0944	0.1096<br>0.0996<br>0.1200	0.0147<br>0.0114<br>0.0189	0.0163<br>0.0125<br>0.0205	0.0226<br>0.0186<br>0.0270	0.0287 ↑<br>0.0236<br>0.0345
	hot & dry	0.0265<br>0.0224<br>0.0260	0.0576<br>0.0487<br>0.0669	0.0777<br>0.0654<br>0.0905	0.0234<br>0.0202<br>0.0267	0.0625<br>0.0517<br>0.0744	0.0846<br>0.0691<br>0.1009	0.0153<br>0.0105<br>0.0228	0.0162<br>0.0104<br>0.0239	0.0161<br>0.0109<br>0.0227	0.0218<br>0.0146<br>0.0310
	cool & wet	0.0236<br>0.0224<br>0.0260	0.0756<br>0.0588<br>0.0942	0.0961<br>0.0745<br>0.1209	0.0208<br>0.0162<br>0.0262	0.0856<br>0.0663<br>0.1089	0.1098 ↑<br>0.0846<br>0.1416	0.0570<br>0.0112<br>0.1725	0.0651<br>0.0118<br>0.1819	0.0412<br>0.0106<br>0.1413	0.0521<br>0.0137<br>0.1784
	hot & wet	0.0239<br>0.0224<br>0.0260	0.0520<br>0.0456<br>0.0591	0.0708 ↓<br>0.0619<br>0.0807	0.0207<br>0.0183<br>0.0232	0.0552<br>0.0479<br>0.0633	0.0750↓<br>0.0647<br>0.0863	0.0138<br>0.0103<br>0.0185	0.0138<br>0.0105<br>0.0184	0.0151<br>0.0115<br>0.0196	0.0205 ↓<br>0.0157<br>0.0267

Supplementary table 2. The posterior mean estimates and 95%-confidence intervals for the model parameters under several seasonality-partitions (rows) and inference scenarios (columns). We use symbols ↓ and ↑ to denote for the minimum and maximum values of the posterior means of infectivity. In every cell, first number is the posterior mean, followed by the lower and upper limits of the confidence intervals.

2 Climate seasons used in the modeling

The different climate partitions are visualized below in Supplementary Figure 2. For the minimum temperature, we considered threshold values 19°C and 23°C, while for the average rainfall, we considered thres holds: 10mm, 75mm and 200mm.

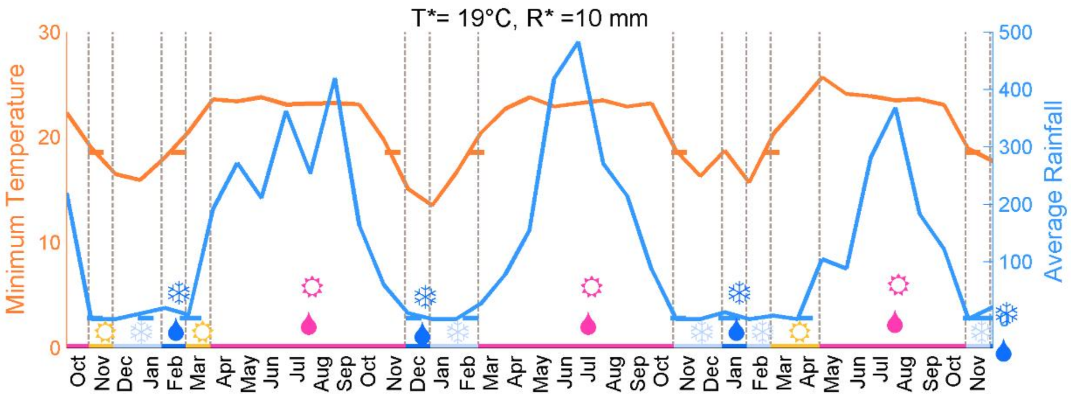

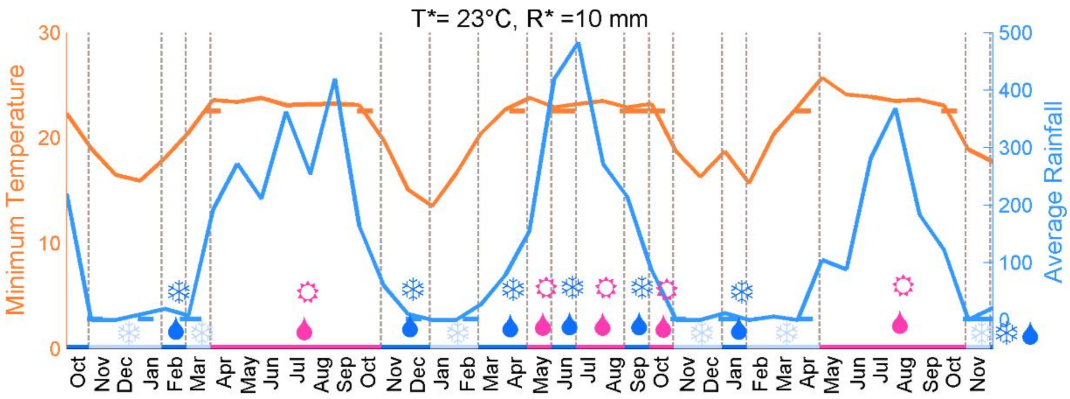
T*= 23°C, R* =10 mm
Minimum Temperature
Average Rainfall
0
10
20
30
0
100
200
300
400
500
Oct Nov Dec Jan Feb Mar Apr May Jun Jul Aug Sep Oct Nov Dec Jan Feb Mar Apr May Jun Jul Aug Sep Oct Nov Dec Jan Feb Mar Apr May Jun Jul Aug Sep Oct Nov

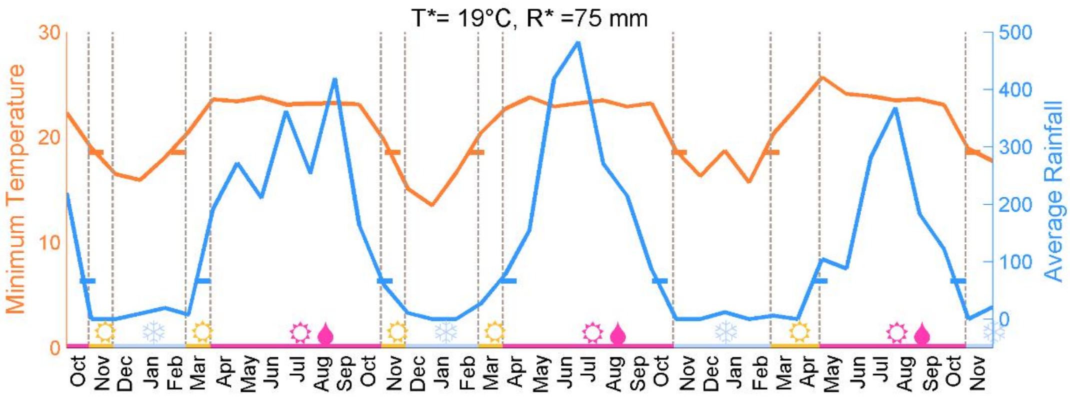
T*= 19°C, R* =75 mm
Minimum Temperature
Average Rainfall
0
10
20
30
0
100
200
300
400
500
Oct Nov Dec Jan Feb Mar Apr May Jun Jul Aug Sep Oct Nov Dec Jan Feb Mar Apr May Jun Jul Aug Sep Oct Nov Dec Jan Feb Mar Apr May Jun Jul Aug Sep Oct Nov

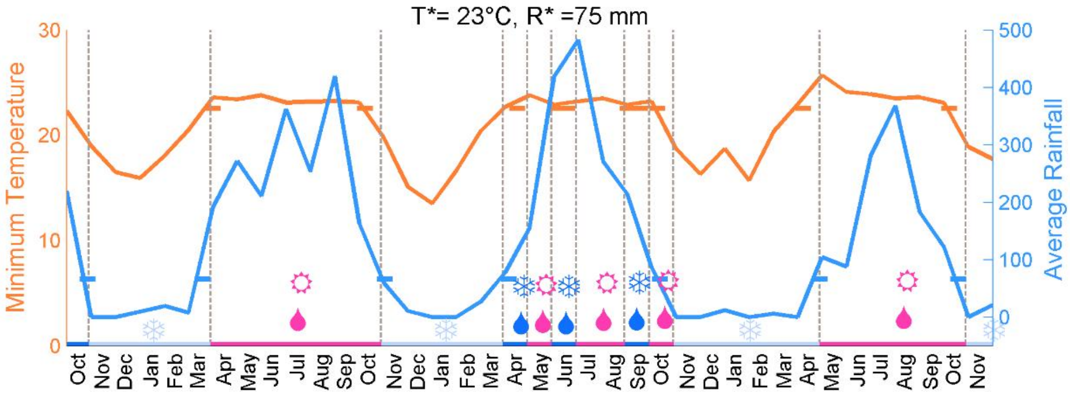
T*= 23°C, R* =75 mm
Minimum Temperature
Average Rainfall
0
10
20
30
0
100
200
300
400
500
Oct Nov Dec Jan Feb Mar Apr May Jun Jul Aug Sep Oct Nov Dec Jan Feb Mar Apr May Jun Jul Aug Sep Oct Nov Dec Jan Feb Mar Apr May Jun Jul Aug Sep Oct Nov

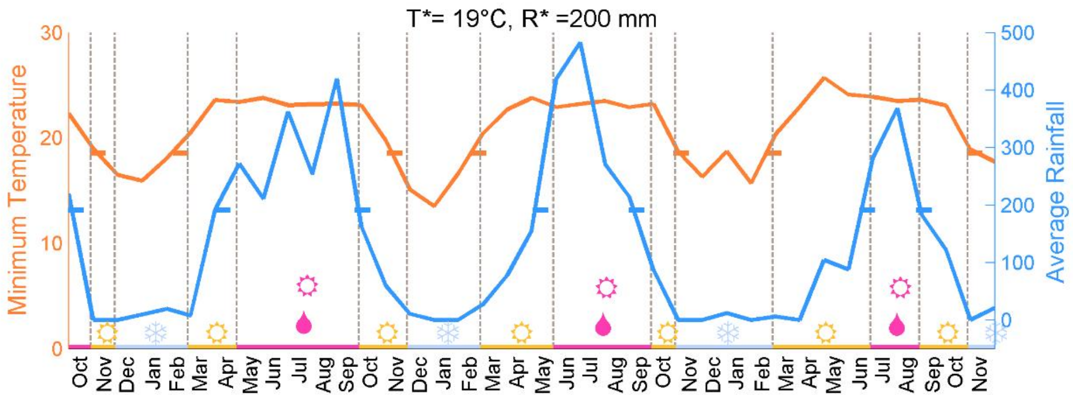

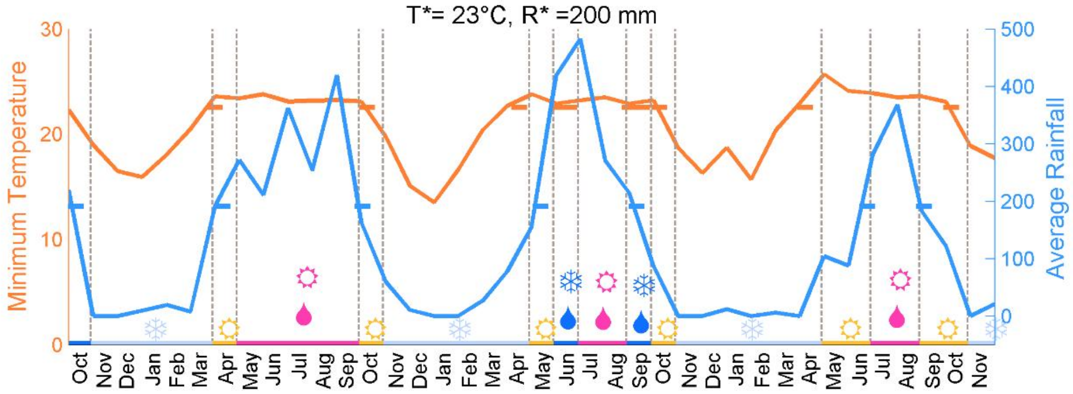

Supplementary Figure 2: Climate characteristics in the Mae Sot region during the study period and the corresponding seasons for six different combinations of threshold values, which are given in the title of each figure. The climate curves visualize the minimum monthly temperature (in Celsius) in orange and average rainfall (in millimeters) in blue. On the horizontal axis the study months are shown, and above each month the color indicates the season the month is assigned to. The seasons are hot (☼), hot and wet (♦☼), cool (❄) and cool and wet (♦❄). The vertical lines show when the seasons change, while the horizontal ticks intersecting with the temperature and rainfall curves show when the corresponding threshold values are exceeded.

3 Model specifications

3.1 The reduced state space of the Markov model for a particular sampling interval

As explained in the main article, we compute the likelihood for each pair of consecutive observations as n-step transition probability, where n equals the number of days between the observation times. For each of the n days, the one-day transition probabilities of the individual are described with matrix M_s (defined in the main article), where s corresponds to the season of the particular day at question.

As seen from Figure 2 and chapter 5.2.1 in the main article, in the colonization model we consider colonizations with different strains as different states of the colonization process. Therefore, due to the extensive number of strains observed in the study population (72 in total), the state space of the full colonization model is large. To be able to calculate the likelihood for a pair of consecutive observations, we consider the same colonization model as explained in the main article but in a reduced state space. The reduced state space is obtained by lumping together very unlikely states into a single state. We explain below the exact states that are lumped together for every pair of consecutive observations.

Notation. We use the notation in which the colonization state of a host i at the sampling time $t^i(j)$ is a duplet $I(t^i(j)) = \{x_1, x_2\}$, where $x_1, x_2 \in \{\emptyset, 1, \ldots, N_s\}$ denote for the labels of colonizing strains, or lack of them, which is denoted with $\emptyset$. To define the reduced state space corresponding to any *pair of consecutive* observations $I(t^i(j-1))$ and $I(t^i(j))$, denote the earlier observed colonization status with $I(t^i(j-1)) = \{f_1, f_2\}$ and the later observed colonization status with $I(t^i(j)) = \{s_1, s_2\}$. Denote with * any strain different from the strains f_1, f_2, s_1, s_2, and denote with $\{*,*\}$ a colonization state in which a host is colonized with two different strains, neither of which equals f_1, f_2, s_1, s_2.

Reduced State space. The reduced state space for a fixed pair of consecutive observations consists of the following states:

$\{\emptyset,\emptyset\}$, $\{f_1,\emptyset\}$, $\{f_2,\emptyset\}$, $\{f_1,f_2\}$,$\{f_1,*\}$,$\{f_2,*\}$, $\{*,\emptyset\}$, $\{*,*\}$, $\{s_1,\emptyset\}$, $\{s_2,\emptyset\}$, $\{s_1,s_2\}\{s_1,*\}$, $\{s_2,*\}$, $\{s_1,f_1\}$, $\{s_1,f_2\}$, $\{s_2,f_1\}$ and $\{s_2,f_2\}$.

Here f_1, f_2, s_1, s_2 are the strains that were actually observed in the consecutive pair of samples from the considered individual. The reduced state space thus consists of the different combinations of the actually observed strains, together with the states ∅ and *, corresponding to being uncolonized and colonized with a new strain, respectively.

Transition probabilities for the lumped states. The transition probabilities of the Markov model in the reduced state space are otherwise as defined in the main article, but we set the transition probabilities considering the lumped states (states with an unobserved strain *) equal to:

current state	end state	probability
{∅, ∅}	{*, ∅}	$q\beta^s$
{x, ∅}	{x, *}	$q\theta\beta^s$
{*, ∅}	{*, *}	$q'\theta\beta^s$
{*, ∅}	{∅, ∅}	γ^s
{*, *}	{*, ∅}	$2\gamma^s$

Supplementary table 3: The transition probabilities of the colonization model, in which certain states are lumped together into a single state.

Above x is any of the f_1, f_2, s_1, s_2. We set the parameter q to equal to the prevalence of strains other than f_1, f_2, s_1, s_2:

$$q = 1 - p(f_1) - p(f_2) - p(s_1) - p(s_2) .$$

For the parameter q' we use the following approximation:

$$q' = 1 - p(f_1) - p(f_2) - p(s_1) - p(s_2) - \boldsymbol{\omega}$$

Additionally, we set $\omega = 0.0058$, which is the median frequency of the strains in the serotype distribution.

Motivation for considering reduced state space. The key point here is to treat separately the states with the strains that were actually observed in the two sampling times, and lump together possible states that consider the unobserved strains. The probability of a transition from the pair {*, Ø} to the pair {*, *}, defined as above is the only approximation that introduces a deviation of the lumped Markov model from the full Markov model with all the strains treated separately. This is because a constant $\boldsymbol{\omega}$ is used instead of explicitly modeling the serotype diversity distribution. While this is reasonable approximation in itself, we also can predict *a priori* that the probability of the chain visiting state {*, *} during the month between the observation times is very low both for the lumped and the full model. This is because the rate of co-colonization is low in general. On the other hand, the sampling is dense enough to detect most of the colonizations, which are known to last approximately at least one month.

3.2 Strain-effects in the biologically detailed model

In the biologically realistic colonization model, the clearance rates are scaled according to the estimates given in Table 2 of the paper by [1]. In this paper, point estimates are given to the rates of clearance for 28 most common serotypes. Based on that information, we construct a serotype-specific modifier to the clearance rates as follows:

$$h(k) = \frac{cl(k)}{median(cl)}$$

Above *cl(k)* is the point estimate in the paper for the clearance rate for strain *k*, and *median(cl)* is the median clearance rate of all the estimated clearance rates. We thus normalize the point estimates for the clearance rates so that the strains with median clearance rate have $h(k) = 1$. For those strains that were not considered in the paper by [1], we set $h(k) = 1$ in our analysis.

We assume that the strain label affects the colonization dynamics for any strain *x* and *y* under the neutral model as follows:

current state	end state	probability
{x, Ø}	{Ø, Ø}	$h(x)\gamma^s$
{x, y}	{x, Ø}	$h(y)\gamma^s$
{*, Ø}	{Ø, Ø}	γ^s
{*, x}	{x, Ø}	γ^s
{*, x}	{*, Ø}	$h(x)\gamma^s$
{*, *}	{*, Ø}	γ^s

As the effect of the strain is multiplicative to the rate of clearance, this means that the effect of season and the label of the colonizing strain to the clearance rate are independent of each other. From this assumption it follows that the relative differences between the clearance rates of different strains during different seasons are the same. Observe that the strain effect of an unobserved strain is set to equal 1.

3.3 Effects of exposures in the biologically detailed model

In the biologically realistic colonization model, we also assume that the colonization history of a host affects the dynamics of the future infections. We denote this colonization history with $I(t^i(1{:}j))$, that is the time series observed from the individual i up until time the sampling time $t^i(j)$. We model the effects of the colonization history with two functions, f and g Given a colonization history $I(t^i(1{:}j))$, the functions take values on the domain of individuals $i \in 1, \dots, N$, strains $x \in 1, \dots, S$, and moments of time t. In essence, f and g describe the multiplicative effects of $I(t^i(1{:}j))$, to the colonization and clearance rates of any strains x and y:

current state	end state	probability
{Ø, Ø}	{x, Ø}	$f(i, x, t^i(j))\beta^s p(x)$
{x, Ø}	{Ø, Ø}	$g\big(i, x, t^i(j)\big)h(x)\gamma^s$

{x, Ø}	{x, y}	$f(i, y, t^i(j))\theta\beta^s p(y)$
{x, y}	{x, Ø}	$g(i, y, t^i(j))h(y)\gamma^s$
{Ø, Ø}	{*, Ø}	$mean\left(f(i, \bullet, t^i(j))\right)\ \beta^s q$
{x, Ø}	{x, *}	$mean\left(f(i, \bullet, t^i(j))\right)\ \theta\beta^s q$
{*, Ø}	{*, *}	$mean\left(f(i, \bullet, t^i(j)\ )\right)\theta\beta^s q'$
{*, Ø}	{Ø, Ø}	$mean\left(g(i, \bullet, t^i(j))\right)\ \gamma^s$
{*, *}	{*, Ø}	$mean\left(g(i, \bullet, t^i(j))\right)\ \gamma^s$

Above $mean\left(f(i, \bullet, t^i(j))\right)$ and $mean\left(g(i, \bullet, t^i(j))\right)$ denote the average value of *f*, (or *g*), in individual i at time $t^i(j)$, taken over all the strains. To define the functions f and g, given time series $I(t^i(1{:}j))$, denote with $S^i_{t(1:j)}$ the set of strains that individual i has been observed to be colonized with up until time $t^i(j)$, but which are not colonizing him at time $t^i(j)$. In other words, $S^i_{t(1:j)}$ describes the set of strains the individual *i* was colonized *and has cleared* before $t^i(j)$. We define function f as follows:

$$f(i, x, t^i(j)) = \begin{cases} c_1, & x \in S^i_{t(1:j)} \\ c_2, & x \notin S^i_{t(1:j)},\ S^i_{t(1:j)} \neq \emptyset \\ 1, & S^i_{t(1:j)} = \emptyset \end{cases}$$

Similarly, function g is defined as:

$$g(i, x, t^i(j)) = \begin{cases} d_1, & x \in S^i_{t(1:j)} \\ d_2, & x \notin S^i_{t(1:j)},\ S^i_{t(1:j)} \neq \emptyset \\ 1, & S^i_{t(1:j)} = \emptyset \end{cases}$$

In short, f and g introduce different effects on the acquisition and clearance rates of any strain x, depending on whether the individual *has never been colonized*, *has been colonized with some strain but not with* x, or if he/she *has been colonized with the strain x* previously. According to our knowledge, currently there does not

exist very precise information on the values of the parameters c_1, c_2, d_1 & d_2. So far it is known from previous studies, that there exists both serotype-specific and serotype-independent immunity that is acquired via past colonizations, but reacquisition of same serotype is perfectly possible. However, it seems that the serotype-specific acquired immunity works mostly by reducing the acquisition rate [2], while the serotype independent immunity works by increasing the clearance rate of future colonizations [3,4].

Based on these previous findings, we set the effects of previous acquisitions to be the following:

parameter	explanation
c_1 = 0.7	Serotype-specific immunity to acquisition
c_2 = 1	Serotype-independent immunity to acquisition
d_1 = 1	Serotype-specific increased clearance rate
d_2 = 1.25	Serotype-independent increased clearance rate

Observe that the above definition of f and g implies that the effects of previous acquisitions are updated in the model only at the observation times, not exactly when the individual manages to clear the infection. As the sampling is dense, this is expected to have a very minor impact on the results.

3.4 Sampling errors

To take the sampling inaccuracy into consideration we define the likelihood contributions of the individuals as:

$$l_i^* = l_i(\beta, \gamma | \{I(t_i)\}) + q \sum_{\{I^\varepsilon(t_i)\}} l_i(\beta, \gamma \mid \{I^\varepsilon(t_i)\}),$$

Where $\{I^\varepsilon(t_i)\}$ denotes for the set of time-series obtained from the actually observed time series $\{I(t_i)\}$ by changing one colonization state to include one additional strain, that was not originally observed at that time,

but was observed before or after that sampling time. Parameter q denotes the probability of missing a serotype, which we set to be 0.2. The current estimates for the accuracy of the swabbing method [5], are typically less than or equal to that. The likelihood for these 'modified' time series is then obtained similarly as explained in equation (2) in the main article. The set $\{I^{\varepsilon}(t_i)\}$ does not cover all the possible alternative colonization histories of individual under imperfect sampling, as there could be colonizing strains that never ended up being observed, or several strains could have been simultaneously missed from the data. However, as the data is not strongly informative on those colonizations, we focused here on taking into account the possibility that an observed colonization epoch was mistakenly assumed shorter than it was in the reality due to inaccurate sampling. The likelihood of missing several strains simultaneously, on the other hand, is low, and such event histories are expected to have decreasing influence on the likelihood surface.

4 Inference

When considering the full data under the neutral model or the biologically realistic model, we used Metropolis-Hastings type MCMC algorithm [6] to obtain a sample from the posterior distribution of the parameters β^s, γ^s for all s. When considering the newborns and their first colonization event, we utilized data-augmentation scheme to sample from the posterior [7]. Details of both methods are explained below.

4.1 The prior distributions

Regardless of the actual partitions of the study months into seasons, we set equal prior distributions for the clearance (and acquisition) rates across seasons. Thus for all $s \in 1, \dots, N_S$, the parameters γ^s have equal prior distributions, and the same holds for all β^s. When considering the neutral model, and when analyzing the first colonization epochs of the newborns, we used the following uniform prior distributions:

$$\gamma^s \sim Uni(0.01, 0.04)$$

$$\beta^s \sim Uni(0.1, 0.2)$$

Under the biologically detailed model, the following prior distributions were used:

$$\gamma^s \sim Uni(0.01, 0.06)$$

$$\beta^s \sim Uni(0.1, 0.2)$$

4.2 The proposal distribution in the MCMC sampler

Denote now with $[\gamma_1, \gamma_2]$ the support of the prior distribution γ^s and with $[\beta_1, \beta_2]$ the support for the prior for β^s, for every s. At each step of the markov chain monte carlo procedure, when the current parameter values are (γ^s, β^s), $s \in 1, \dots, N_s$, the proposed new parameters $\widetilde{\gamma^s}, \widetilde{\beta^s}$ for all the seasons $s \in 1, \dots, N_s$ are sampled from:

$$\widetilde{\gamma^s} \sim Uni([\gamma^s - \varepsilon_\gamma, \gamma^s + \varepsilon_\gamma] \cap [\gamma_1, \gamma_2]), \qquad s \in 1, \dots, N_s$$
$$\widetilde{\beta^s} \sim Uni([\beta^s - \varepsilon_\beta, \beta^s + \varepsilon_\beta] \cap [\beta_1, \beta_2]), \qquad s \in 1, \dots, N_s \qquad 1$$

Thus, for each of the seasons s the proposed new rates are sampled uniformly in the ε_γ or ε_β – neighborhood of the current value of the rate, truncated at the support of the prior distribution. The parameters were accepted and rejected by the typical Metropolis-Hastings step. Uniform proposal distribution allowed for fast evaluation of the acceptance probability of the proposed new value. The actual values for ε_γ and ε_β were set based on preliminary MCMC runs, monitoring manually the acceptance rate and the mixing. We give the values used in a table shown in the following section.

4.3 Details of the MCMC runs

For each of the inference scenarios we run 20 independent MCMC chains, initiated at different points in parameter space, which were sampled from the prior. Posterior samples are obtained by merging the samples in the different chains, from which the burn-in samples were first discarded. To ensure the convergence, Gelman-Rubin statistics were computed, using the samples from the 20 chains, from which the samples from the burn-in period are discarded. We considered the chains to have converged, when the statistic was below

1.1 for all of the model parameters. In the Supplementary table 4 below we list the details of the MCMC runs, including proposal distributions used, the length of burn-in period and the resulting Gelman-Rubin statistics corresponding to different inferences made in our analysis.

Inference scheme	Season partition	proposal distribution	burn-in	length of chain after burn-in	interval-based Gelman-Rubin Statistic
Neutral model	Months as seasons	ε_γ = 0.003<br>ε_β = 0.003	2000	17 000	between 1.0015 and 1.0492
	T*= 19<br>R*=75	ε_γ = 0.0008<br>ε_β = 0.0018	2000	13 000	between 0.9988 and 1.0082
	T*= 23<br>R*=75	ε_γ = 0.0008<br>ε_β = 0.0018	2000	10 000	between 0.9964 and 1.0343
	T*= 19<br>R*=10	ε_γ = 0.0008<br>ε_β = 0.0018	2000	10 000	between 0.9979 and 1.0488
	T*= 23<br>R*=10	ε_γ = 0.0008<br>ε_β = 0.0018	2000	10 000	between 0.9980 and 1.0138
	T*= 19<br>R*=200	ε_γ = 0.0008<br>ε_β = 0.0018	2000	8000	between 0.9983 and 1.0091
	T*= 23<br>R*=200	ε_γ = 0.0008<br>ε_β = 0.0018	2000	8000	between 0.9992 and 1.0453
Biologically realistic model<br><br>(strain effects and effects of the past exposures)	Months as seasons	ε_γ = 0.004<br>ε_β = 0.004	2000	18000	between 0.9965 and 1.0419
	T*= 19<br>R*=75	ε_γ = 0.0008<br>ε_β = 0.0018	2000	8000	between 1.0013 and 1.0129
	T*= 23<br>R*=75	ε_γ = 0.0008<br>ε_β = 0.0018	2000	8000	between 0.9960 and 1.0065
	T*= 19<br>R*=10	ε_γ = 0.0008<br>ε_β = 0.0018	2000	8000	between 0.9960 and 1.0065
	T*= 23<br>R*=10	ε_γ = 0.0008<br>ε_β = 0.0018	2000	8000	between 0.9960 and 1.0041
	T*= 19<br>R*=200	ε_γ = 0.0008<br>ε_β = 0.0018	2000	8000	between 1.0009 and 1.0156
	T*= 23<br>R*=200	ε_γ = 0.0008<br>ε_β = 0.0018	2000	8000	between 0.9981 and 1.0827

Supplementary Table 4: Details of the MCMC chains that were run to sample from the posterior distribution.

4.4 Trace plots

As examples we show some of the trace plots that also visually indicate adequate convergence to the posterior distribution.

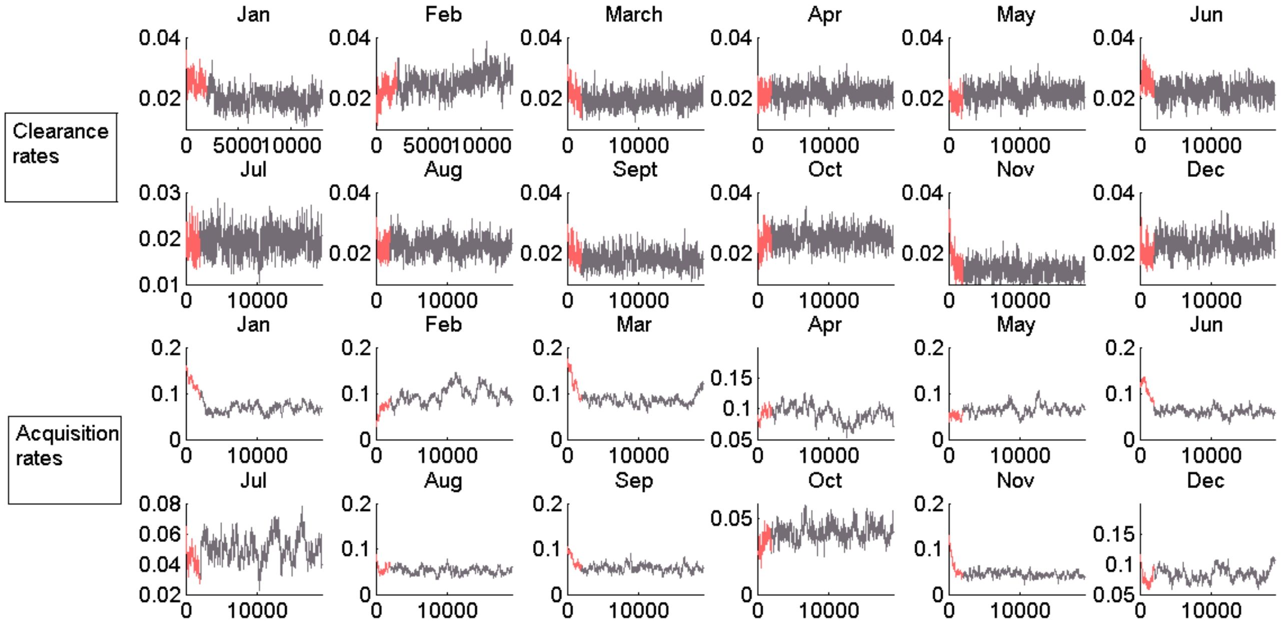

Supplementary Figure 3: Trace plots of one mcmc-run of 19000 steps, in which calendar months were considered as seasons. The panels visualize the sampled values against the MCMC step. The first 12 panels visualize the month-specific clearance rates from January to September, and the next 12 panels visualize the parameter values for the month-specific clearance rates. The first 2000 steps, visualized in red color, were considered as burn-in, and those samples were discarded from the posterior sample.

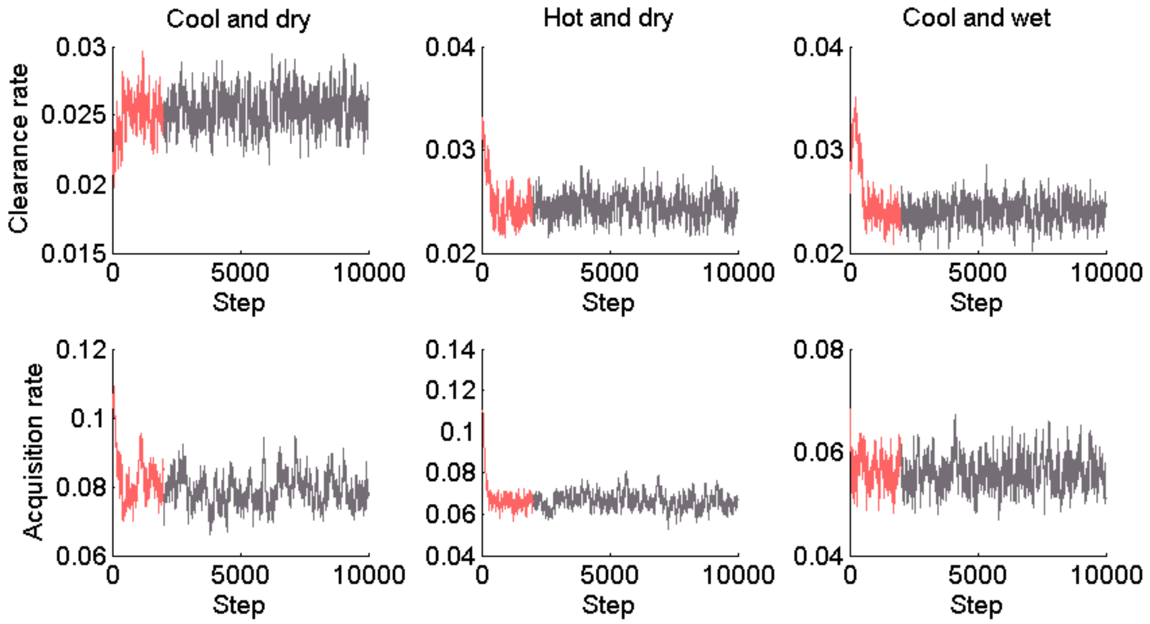

Supplementary Figure 4: The trace-plots showing the sampled parameter values when seasons were the ones corresponding to a threshold values R*= 200 mm and T*=19C, and the inference scheme corresponded to that of a neutral model. First three panels depict the clearance rate parameters for the three climates, and the next three depict the acquisition rate for the same three climates. The first 2000 samples, visualized in red, were considered as burn in, and discarded from the actual posterior sample.

4.5 Inference considering only the first colonization event of the newborns

The likelihood for β_n^s (n for newborns) can be written as:

$$P(\beta_n^s \,|D) = \int P(\beta_n^s|\, t^*) P(t^*|D)\, dt^* \qquad (2)$$

As explained in the main article, the probability $P(\beta_n^s|\, t^*)$ is analytically tractable Beta distribution, once the infection times t* for every individual are set. By sampling enough many random realizations of t*, from the data augmentation distribution, denoted as $P(t^*|D)$, the integral in (2) is approximated.

We set $P(t^*|D)$ such that for all the individuals we pick uniformly at random $t^*(i)$ from the interval $[t_l(i), t_c(i)]$, where $t_c(i)$ denotes for the first time the individual i was observed to be colonized, and

$t_l(i)$ denotes for the last sampling time before that (i.e. the most recent time when it was not yet observed to be colonized). In practice, we approximated the distribution in (2) by performing the following sampling:

For every season *s* repeat:

1. Sample *N* realizations of t*
2. Sample *M* parameters β_n^s from the prior distribution.
3. Give each of the *M* parameters a weight, that is $w = \frac{1}{N} \sum_{t*} P(\beta_n^s | \, t^*)$
4. Resample the parameters β_n^s according to weights to obtain the posterior distribution.

In our calculations, we used *N*=*M*=5000. Estimation of the clearance rate in the first colonization epoch of an individual is done similarly. This time the unknown event time is the time of clearance of the strain that was observed colonizing the individual first. Now the unknown event time is sampled from the interval $[t_s(i), t_{\neg s}(i)]$, where $t_s(i)$ denotes for the time the individual was last seen colonized by strain *s*, where s is the first strain it was colonized with, and $t_{\neg s}(i)$ is the first sampling time after $t_s(i)$. If the strain effects for the clearance rate were considered, then the number of days an individual spent colonized with a strain at a certain season was multiplied with the strain-specific modifier (supplementary chapter 3.6) and rounded to the nearest integer.

5 Monthly birth rates in the cohort

The babies included in the study were selected independently of the month at which they were born. In detail, between October 2007 and November 2008, all the pregnant women in the camp attending the SMRU antenatal clinic at 28–30 weeks gestation, were invited to consent to their infant's participation in a pneumonia cohort study. The mothers were subsequently randomized into a cohort that was sampled each month, and into a cohort that was not sampled systematically each month. The latter cohort was excluded from the seasonality analysis presented here.

Supplementary Figure 5 we shows the distribution for the months of birth for the babies in both two cohorts. Approximately 80% of the women to [8] are estimated to attend the SMRU antenatal clinic, so the data covers the majority of the births in the camp during that time period, and the distribution could reflect the actual distribution of the births. A peak in child births is observed in December 2007, when 114 babies were born, compared to the 51 babies born in July 2008. We also show the distribution of birth months for the subset of infants, which were studied each month, and who were also the cohort that was analysed in the current study.

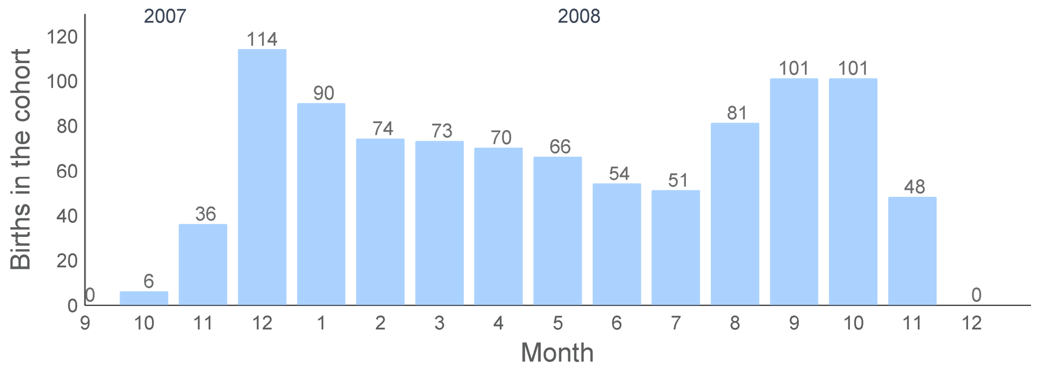

Supplementary Figure 5: The numbers of births in different months during the onset of the study, 2007-2008. The dates of birth cover the cohort that was sampled each month, and the cohort that was sampled infrequently.

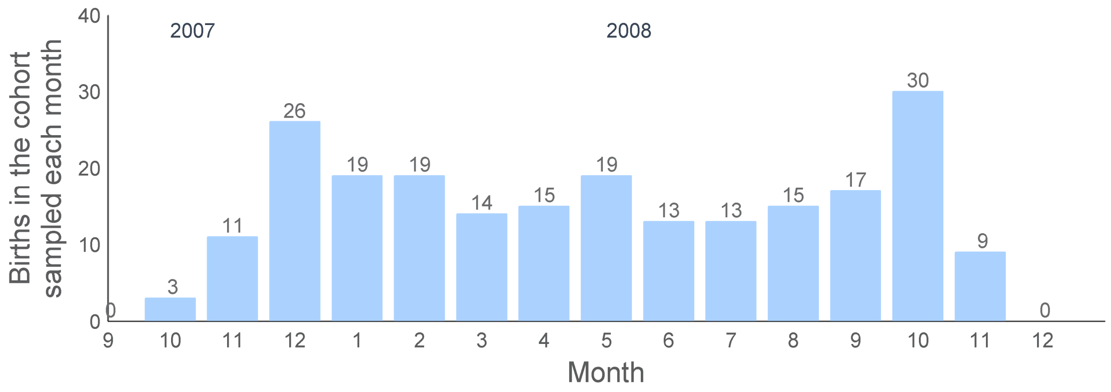

Supplementary Figure 6: The numbers of births in different months in the cohort that was sampled each month. This is the cohort that was analysed in the current analysis.

6 The minimum-, mean and maximal temperatures during the study

Supplementary Figure 6 shows the minimum, mean and maximum monthly temperatures in the study region during the considered years. The three quantities are observed to correlate, and the curve for minimum temperature appears most stable.

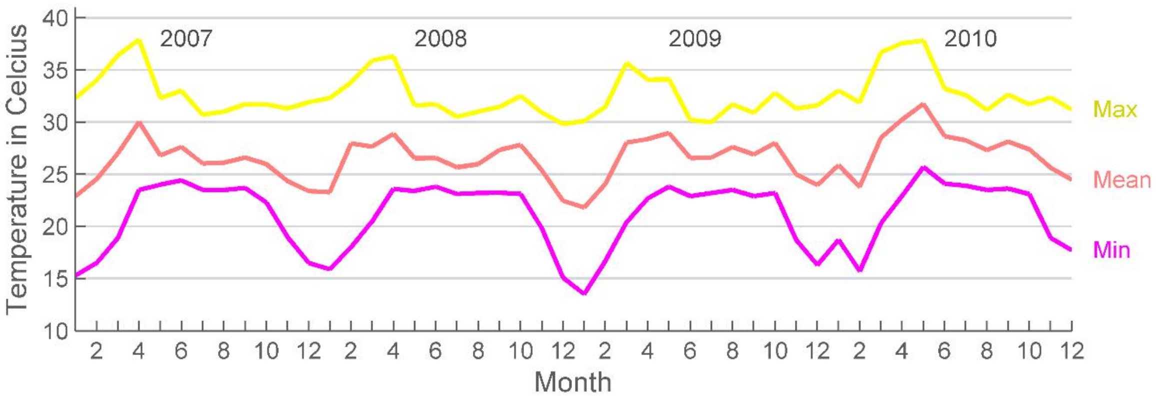

Supplementary Figure 7: The measured maximum, mean and the minimum temperatures in the Mae Sot region during the study.

7 Supplementary References

1 Abdullahi, O. *et al.* Rates of acquisition and clearance of pneumococcal serotypes in the nasopharynges of children in Kilifi District, Kenya. *The Journal of infectious diseases* **206**, 1020-1029, doi:10.1093/infdis/jis447 (2012).

2 Weinberger, D. M. *et al.* Epidemiologic evidence for serotype-specific acquired immunity to pneumococcal carriage. *Journal of Infectious Diseases* **197**, 1511-U1524 (2008).

3 Malley, R. *et al.* CD4(+) T cells mediate antibody-independent acquired immunity to pneumococcal colonization. *P Natl Acad Sci USA* **102**, 4848-4853 (2005).

4 Lu, Y. J. *et al.* Interleukin-17A mediates acquired immunity to pneumococcal colonization. *PLoS pathogens* **4**, e1000159, doi:10.1371/journal.ppat.1000159 (2008).

5 Satzke, C. *et al.* Standard method for detecting upper respiratory carriage of Streptococcus pneumoniae: updated recommendations from the World Health Organization Pneumococcal Carriage Working Group. *Vaccine* **32**, 165-179, doi:10.1016/j.vaccine.2013.08.062 (2013).

6 Hastings, W. K. Monte-Carlo Sampling Methods Using Markov Chains and Their Applications. *Biometrika* **57**, 97-& (1970).

7 Tanner, M. A. & Wing, H. W. The Calculation of Posterior Distributions by Data Augmentation. *J Am Stat Assoc* **82**, 528-540 (1987).

8 Turner, C. *et al.* A three year descriptive study of early onset neonatal sepsis in a refugee population on the Thailand Myanmar border. *BMC infectious diseases* **13**, doi:Doi 10.1186/1471-2334-13-601 (2013).
